# Adsorption and Catalytic Debromination of 2,6-Dibromophenol on Ni(111): A First-Principles Mechanistic Study

**DOI:** 10.1021/acs.jpcc.6c03510

**Published:** 2026-09-09

**Authors:** Paavo Auvinen, Mikko Linnolahti, Ville H. Nissinen, Mika Suvanto, Janne Jänis, Jarkko J. Saarinen

**Affiliations:** Department of Chemistry and Sustainable Technology, 163043 University of Eastern Finland, P.O. Box 111, Joensuu FI-80101, Finland

## Abstract

Catalytic debromination is increasingly relevant for environmental remediation and the recycling of plastics containing brominated flame retardants, yet atomistic understanding of the underlying surface chemistry on transition-metal catalysts remains limited. Density functional theory calculations were used to investigate the adsorption and surface-mediated debromination of 2,6-dibromophenol on Ni(111), with 2-bromophenol and phenol included for systematic comparison. Bromine substitution was found to fundamentally alter the adsorption mode: while phenol preferentially chemisorbs at ∼2.2 Å from the surface, 2,6-dibromophenol is more stable in a physisorbed configuration at ∼3.5 Å, with 2-bromophenol at the crossover. This reversal originates from the enhanced polarizability of the C–Br bonds, which strengthens long-range dispersion sufficiently to outweigh the covalent stabilization available in close-coordination geometries. The effect is captured by BEEF-vdW and vdW-DF but missed by PBE, placing a methodological requirement on DFT studies of halogenated aromatic adsorption. Reaction pathway analysis identifies a stepwise debromination-hydrogenation sequence as the preferred route, with ring hydrogenation (0.56 eV barrier) rather than C–Br cleavage (0.16 eV) as the rate-limiting step. Direct hydrogenolysis (0.77 eV) and sequential debromination prior to hydrogenation are both disfavored. The dominant practical limitation is not the activation of the C–Br bond but the strong binding of the released bromine on the surface (−1.45 eV), which blocks active sites and rationalizes the experimental need for halogen scavengers or regeneration strategies. The findings provide an atomistic foundation for understanding the catalytic dehalogenation of polybrominated aromatics, including tetrabromobisphenol A, on earth-abundant transition-metal catalysts.

## Graphical abstract

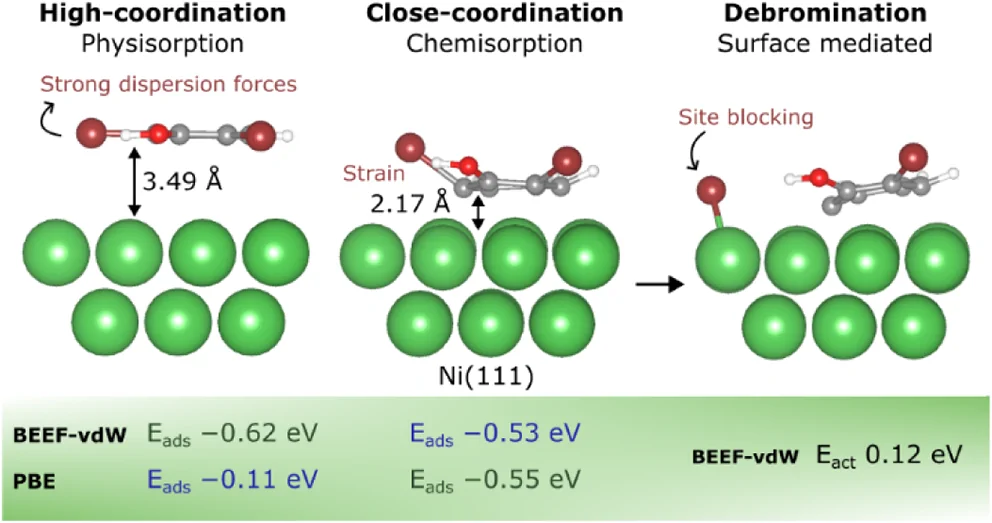

## 1 Introduction

Recycling plastic waste is becoming increasingly important for producing sustainable resources and advancing a circular economy. Mechanical recycling aims to convert the plastic waste into materials and products without significantly changing the chemical structure or quality, whereas chemical recycling focuses on producing valuable chemicals through depolymerization processes.^1,2^ Currently, only a limited number of polymer types are routinely recycled. For instance, polyethylene and polyethylene terephthalate account for the majority of recycled commodity plastics in the United States, while only small fractions of polypropylene and polystyrene are recycled.^3^ Consequently, many engineering plastics, including high-impact polystyrene and acrylonitrile-butadiene-styrene, remain largely underutilized in existing recycling schemes. A major obstacle to their recycling is the presence of hazardous additives.

Brominated flame retardants (BFRs) are widely incorporated into engineering plastics to enhance fire safety by delaying ignition.^4,5^ However, these additives pose significant risks to the environment and human health, which severely limits the recyclability of BFR-containing materials. Hence, removal of BFRs from the polymer substrate is a prerequisite for recycling. Various extraction techniques have been developed for this purpose.^6,7^ More recently, catalytic debromination, typically achieved through reductive pathways, has emerged as a promising alternative. It both reduces the environmental persistence of BFRs and enables their transformation into platform chemicals.^8,9^

Noble-metal catalysts frequently exhibit high activity for debromination.^8–11^ From the perspective of plastic-waste valorization, replacing noble metals with earth-abundant alternatives represents a more sustainable and economically viable approach. Nickel has consequently attracted attention as a promising candidate with demonstrated catalytic performance.^12–14^ While experimental studies on catalytic debromination have steadily increased, theoretical investigations have received comparatively less attention. Studies addressing BFR adsorption and surface-mediated debromination in particular remain scarce, whereas gas-phase decomposition studies of certain BFRs, such as tetrabromobisphenol A (TBBPA), have been reported.^15,16^

Beyond plastics recycling, catalytic dehalogenation has potential applications in environmental processes, such as wastewater treatment and the elimination of per- and polyfluoroalkyl substances (PFAS).^17,18^ Given the increasing relevance of dehalogenation, the present work employs first-principles DFT calculations to investigate debromination pathways of the most widely used flame retardant, TBBPA, on nickel surface, using 2,6-dibromophenol (DBP) as a representative model compound. The first part of the work comprises a systematic DFT investigation of the adsorption behavior of DBP, 2-bromophenol (BP), and phenol on the most stable low-index surface of nickel, Ni(111). The second part focuses on the elementary reaction steps and mechanisms governing catalytic debromination. Together, the results provide an atomistic picture of the adsorption behavior of brominated aromatics and surface-mediated debromination on nickel, offering atomistic insight into guide future studies.

## 2 Computational Methods

Calculations were performed with the GPAW software using the projector augmented wave (PAW) formalism. Ni(111) surfaces were constructed from an optimized FCC unit cell with a lattice parameter of 3.53 Å. Surface calculations employed 4-layer slabs with the two bottom layers fixed at their optimized positions. All models were periodic in the directions parallel to the surface, and a 15 Å vacuum was included perpendicular to the slab. A plane-wave basis with a 450 eV cutoff energy, a 2 × 2 × 1 Monkhorst–Pack k-point mesh, and 0.01 eV Fermi smearing were used for all surface calculations. Increasing k-point mesh to 3 × 3 × 1 changed the adsorption energies of DBP, BP and Ph by less than 0.05, 0.04, and 0.07 eV, respectively. Extending the slab from four to five Ni layers changed the DBP adsorption energy by 0.03 eV, and increasing vacuum spacing from 15 to 20 Å changed it by 0.01 eV. The BEEF-vdW^19^ functional was used to screen adsorption geometries, while PBE^20^ and vdW-DF^21^ were employed as reference functionals for the most stable structures. The DFTD-D3 dispersion correction with Becke-Johnson-damping was applied on PBE-optimized geometries using the implementation by Grimme et. al.^22,23^ Gas-phase TBBPA, DBP, BP and Ph were optimized with otherwise identical parameters, but in a cell with doubled lattice vectors and a 1 × 1 × 1 Monkhorst–Pack mesh. Projected density of states (PDOS) calculations employed an 8 × 8 × 1 mesh and 0.1 eV Fermi smearing. Small gas-phase molecules (HBr, Br_2_, H_2_) were optimized with original cell dimensions and parameters. All calculations were spin-polarized to account for the ferromagnetic nature of nickel.

Structure optimizations were performed with the FIRE or BFGS algorithms. Local minima were relaxed until the largest force was equal to or below 0.03 eV Å^–1^. Transition states (TS) and minimum energy paths were computed with the nudged elastic band (NEB) method. Calculations used 5–7 images depending on the reaction. NEB and scan calculations were performed with a force threshold of 0.05 eV Å^–1^. Bader charge analysis was performed using the code provide by the Henkelman group,^24^ and charge density differences (CDD) were obtained from GPAW output and visualized with VESTA.^25^

Vibrational analyses on selected structures were performed using a partial Hessian approach, in which only the adsorbate atoms were displaced while the nickel slab atoms were kept fixed following the common practice for extended surface calculations to reduce computational cost. Examination of the corresponding vibrational modes indicated that these imaginary frequencies originated from artifacts of the partial Hessian approach (e.g., hydroxyl rotation) and did not reflect structural instabilities.

Adsorption energies in this work are reported as electronic energies. Adsorption energy was calculated with eq 1, where E_ads_ is the electronic adsorption energy, E_slab+molecule_ is the electronic energy of a molecule adsorbed on Ni(111), E_slab_ is the electronic energy of the clean Ni(111) with two bottom layers fixed, and E_molecule_ is the electronic energy of the optimized gas-phase molecule.

1 $$E_{ads}=E_{slab+molecule}-E_{slab}-E_{molecule\left(g\right)}$$

Reaction energies were calculated relative to a common reference state consisting of the clean Ni(111) slab plus the gas-phase DBP molecule. All reaction path-energies include zero-point energy (ZPE) corrections. At each reaction path, the total atomic composition was conserved by including gas-phase molecules and/or adsorbed species (e.g., gas-phase H_2_ and surface bound Br after C–Br cleavage) as appropriate.

ZPEs were evaluated using the thermochemistry modules of the Atomic Simulation Environment.^26^ HinderedThermo class was used to evaluate entropic contributions for physisorbed configurations where translational and rotational modes are treated as frustrated modes on the surface. For this, translation and rotation barriers were set to 0.05 and 0.10 eV, respectively. The site density was defined based on one adsorbate per unit cell area. Imaginary frequencies and vibrational modes below 50 cm^–1^ were excluded from the ZPE and entropy calculations to remove artificial contributions from harmonic treatment of anharmonic modes.^27^

Rate constants were computed from activation energies based on the Eyring eq 2, where *k*_b_ is the Boltzmann constant, h is the Planck constant, T is the temperature, R is the ideal gas constant, ΔS^‡^ is the activation entropy, and *E*_a_ is the ZPE corrected activation energy. The activation entropy ΔS^‡^ was assumed to be negligible, given that the elementary steps occur between surface-bound species with limited translational and rotational freedom. The reported rate constants are therefore intended for relative comparison among the elementary steps under a consistent set of assumptions, rather than as absolute predictions of catalytic turnover.

2 $$k=\left[\frac{k_{b}T}{h}e^{\Delta S^{‡}/R}\right]e^{-E_{a}/RT}$$

## 3 Results and Discussion

### 3.1 Adsorption Studies

#### 3.1.1 Dibromophenol

Adsorption of DBP was systematically screened on Ni(111), the most stable low-index surface termination for nickel, which serves here as a mechanistic model of the supported Ni/Al_2_O_3_ catalysts examined experimentally. DBP was chosen as a representative model compound for TBBPA based on the experimental observation that TBBPA undergoes preferential debromination of one aromatic ring at a time on Ni/Al_2_O_3_.^14^ While DBP cannot reproduce the steric and electronic environment imposed by the bridging quaternary carbon of TBBPA, it captures the essential reactivity of the brominated aromatic ring and offers a computationally tractable model for systematic mechanistic analysis. Gas-phase DFT calculations confirmed that the C–Br bond-dissociation energy at the corresponding ortho-positions of TBBPA and DBP differed by less than 0.01 eV. Figure 1 presents the eight adsorption configurations considered, spanning four high-symmetry sites: the bridge site (bri) with two-fold local symmetry, the face-centered cubic (FCC) and hexagonal close-packed (HCP) hollow sites with three-fold symmetry, and the atop site with six-fold symmetry. Geometry optimizations from initial atop close-coordination geometries consistently relaxed the adsorbate to more stable sites for all three studied compounds, and the atop site was therefore identified as unstable and was not considered further. Upon adsorption, the carbon atoms of an aromatic ring can lie between nickel atoms (green in Figure 1), share a nickel atom with a neighboring carbon (blue), or sit directly above a single nickel atom (red). The aromatic ring can orient itself with the C–C bond either aligned along the rows of nickel atoms (0° configuration) or rotated 30° relative to them (30° configuration). Previous DFT simulations have shown that benzene preferentially adsorbs on (111) surfaces in the 30° configuration at bridge sites (bri30).^30–32^ Substitution of hydrogen by functional groups reduces molecular symmetry, giving rise to additional adsorption geometries and an increased number of possible binding modes.^30^

**Figure 1:**
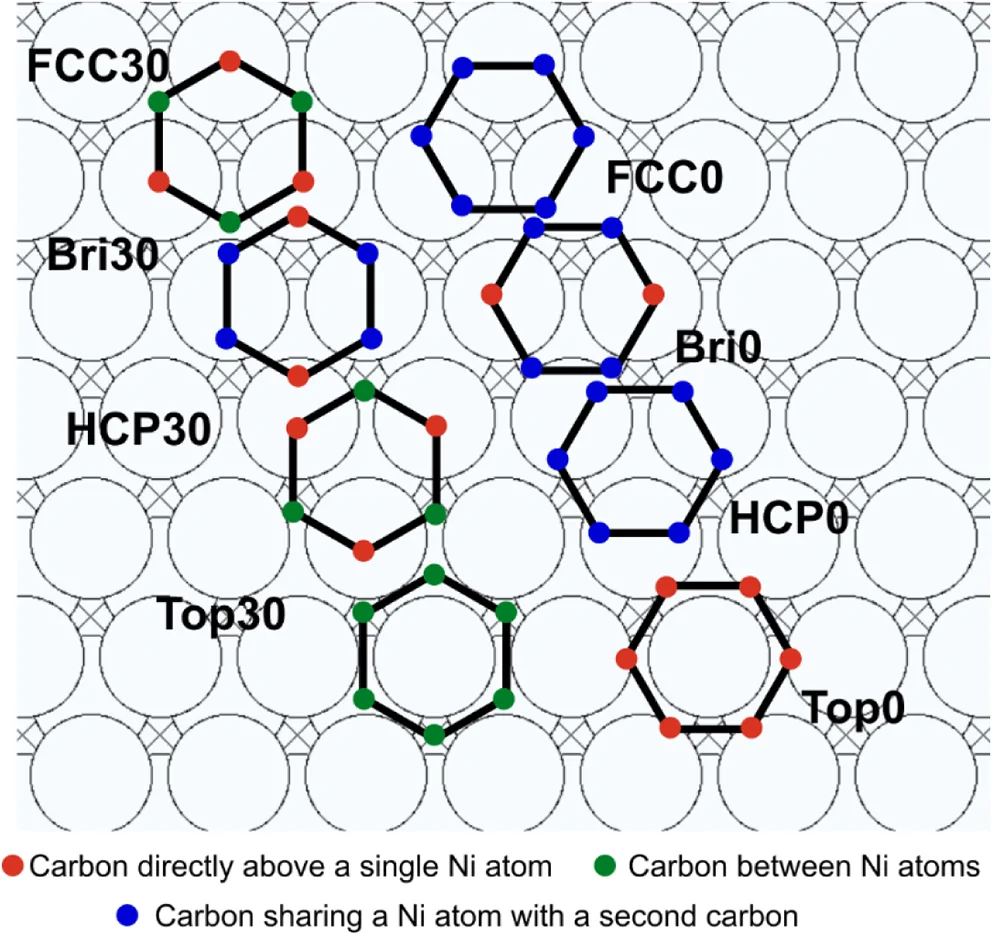
Eight adsorption configurations considered on the Ni(111) surface, spanning four high-symmetry sites (atop, bridge (bri), and the FCC and HCP hollow sites), each tested with the aromatic ring in both 0° and 30° orientations. Color coding indicates the position of each ring carbon relative to the underlying nickel atoms, as defined in the figure key.

##### 3.1.1.1 Physisorption Regime

BEEF-vdW simulations revealed that DBP preferentially coordinated at a relatively large distance from the Ni(111) surface, with average Ni–C separation of 3.49 Å above the bridge site. Typical stable adsorption geometries of aryl adsorbates involve considerably shorter distances, such as 1.99–2.08 Å reported for benzene.^31^ Figure 2a presents the optimized structure of DBP in this high-coordination geometry above the bridge site, with associated binding energy of −0.62 eV. The C–Br bond lengths are 1.917 Å and 1.926 Å, with the bond directed toward the OH group being slightly elongated. These values are essentially identical to the corresponding gas-phase bond lengths of 1.915 Å and 1.927 Å. The C–C bond lengths within the ring likewise remain equal to those of gas-phase DBP, indicating that coordination at this height did not induce significant structural distortion. A reference geometry optimization with the original vdW-DF functional yielded slightly stronger binding (by ∼0.1 eV) relative to BEEF-vdW, consistent with its reported tendency to overestimate long-range interactions.^33–35^ By contrast, the commonly employed PBE functional yielded a substantially weaker adsorption energy of −0.11 eV. Because PBE lacks the non-local correlation terms required to describe long-range dispersion interactions, the result indicates that the stabilization of DBP arises predominantly from van der Waals forces, consistent with physisorption. Applying the D3 correction to PBE-optimized geometry increased the adsorption energy to −1.13 eV, confirming that the stabilization in this geometry is dispersion-dominated.

**Figure 2:**
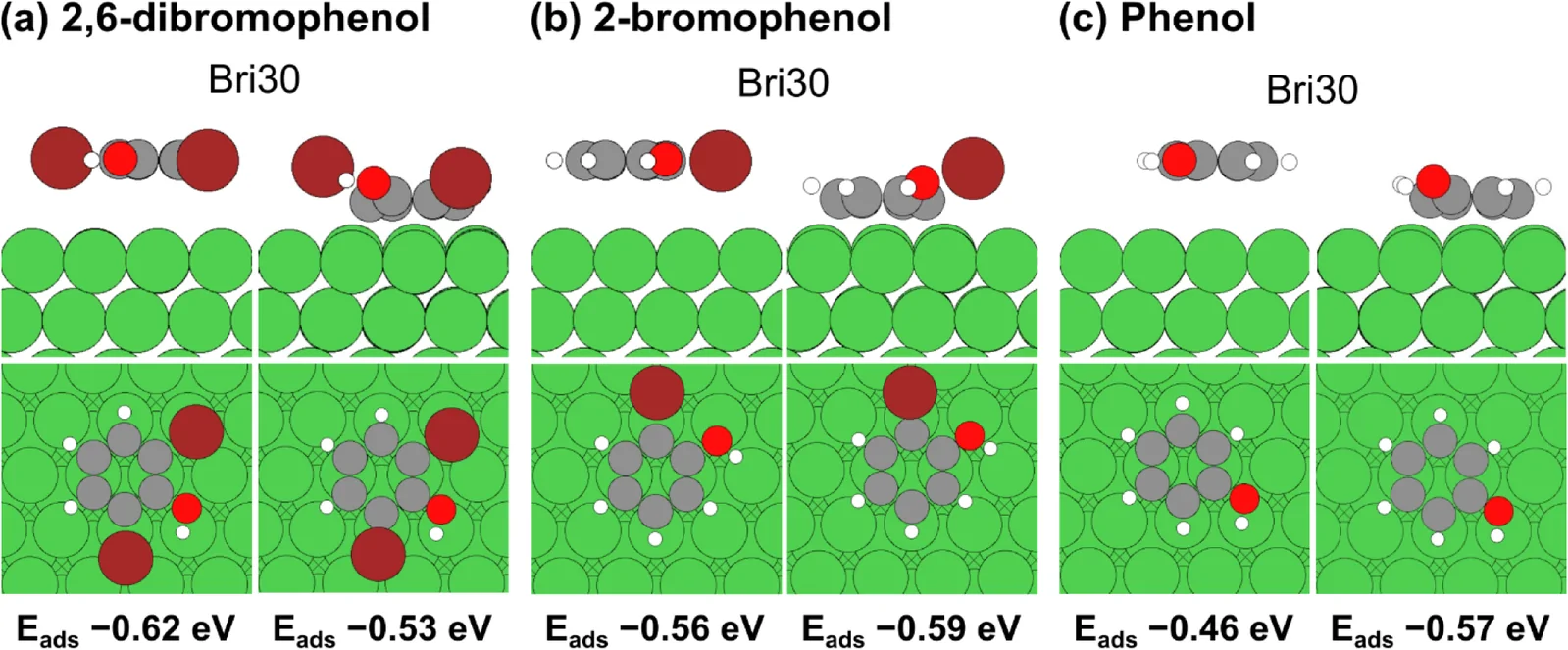
Most stable adsorption geometries on the Ni(111) surface for (a) 2,6-dibromophenol (b) 2-bromophenol, and (c) phenol, optimized with BEEF-vdW functional. For each adsorbate, the high-coordination geometry of the physisorption regime is shown on the left and the close-coordination geometry of the chemisorption regime on the right, each in top (upper) and side (lower) views. Adsorption energies are indicated below each configuration. Color scheme: green = nickel, gray = carbon, red = oxygen, white = hydrogen, dark red = bromine.

Rotation of the aromatic ring about the surface normal, yielding 0° and 30° configurations, as well as variations in the orientation of the substituents relative to surface atoms, did not significantly influence the adsorption energy in the physisorption regime. With the BEEF-vdW functional, the standard deviation among the four tested orientations at the bridge site was only 0.01 eV. Adsorption energies also varied only slightly between different adsorption sites, with a standard deviation of 0.02 eV (Table 1). BEEF-vdW predicted the most stable configuration to be one in which the aromatic ring remained parallel to the Ni(111) surface. Tilting the bromine and hydroxyl substituents toward the surface destabilized the adsorption energy by 0.04 eV, while PBE yielded practically identical adsorption energies for the tilted and parallel geometries.

**Table 1:** Adsorption Energies (eV) of 2,6-Dibromophenol, 2-Bromophenol, and Phenol on Ni(111), Calculated with the BEEF-vdW, vdW-DF, and PBE functionals^a^,^b^

				**Adsorbate**						
		**2,6-Dibromophenol**			**2-Bromophenol**			**Phenol**		
	Site	BEEF-vdW (eV)	vdW-DF (eV)	PBE (D3) (eV)	BEEF-vdW (eV)	vdW-DF (eV)	PBE (D3) (eV)	BEEF-vdW (eV)	vdW-DF (eV)	PBE (D3) (eV)
High-coordination	Bri30	–0.62	–0.72	–0.11 (−1.13)	–0.56	–0.65	–0.13 (−0.97)	–0.46	–0.55	–0.12 (−0.79)
	FCC30	–0.63	–0.72	–0.12 (−1.13)	–0.57	–0.65	–0.15 (−0.99)	–0.47	–0.55	–0.12 (−0.78)
	HCP30	–0.63	–0.73	–0.12 (−1.13)	–0.58	–0.66	–0.14 (−0.99)	–0.48	–0.56	–0.13 (−0.80)
	Top30	–0.59	–0.69	–0.08 (−1.09)	–0.53	–0.61	–0.11 (−0.94)	–0.44	–0.51	–0.09 (−0.75)
Close-coordination	Bri0	–0.09	0.17	–0.09 (−1.60)	–0.32	–0.06	–0.50 (−1.88)	–0.33	–0.02	–0.56 (−1.67)
	Bri30	–0.53	–0.24	–0.55 (−2.08)	–0.59	–0.32	–0.74 (−2.06)	–0.57	–0.30	–0.80 (−1.90)
	FCC0	–0.31	–0.07	–0.35 (−1.93)	–0.46	–0.19	–0.62 (−1.97)	–0.51	–0.24	–0.72 (−1.80)
	FCC30	–0.32	–0.25	–0.48 (−2.01)	–0.39	–0.14	–0.50 (−1.84)	–0.45	–0.24	–0.63 (−1.76)
	HCP0	–0.35	–0.08	–0.41 (−1.97)	–0.49	–0.19	–0.63 (−1.99)	–0.55	–0.24	–0.76 (−1.85)
	HCP30	–0.37	–0.10	–0.38 (−1.92)	–0.45	–0.18	–0.57 (−1.91)	–0.49	–0.24	–0.70 (−1.82)
	Top0	Unstable	-	-	Unstable	-	-	Unstable	-	-
	Top30	Unstable	-	-	Unstable	-	-	Unstable	-	-

a D3 in parentheses refers to DFT-D3 correction.

b “Unstable” denotes configurations that migrated to a different site.

Electronic structure analyses confirmed that DBP binding in the high-coordination geometry is dispersion-dominated rather than covalent. The CDD analysis presented in Figure 3a and Figure S1a showed polarization-driven charge redistribution for every functional used. With the vdW functionals, electron density was depleted from the π-system of the aromatic ring and the bromine p-orbitals, while accumulation occurred on the Ni d-states and intramolecular bonding regions of DBP. The resulting interface dipole, combined with the large Ni–C separation, is characteristic of physisorption.^36^ PBE did not exhibit well-defined d-orbital-like features in the CDD map, instead yielding a more diffuse and delocalized charge redistribution indicative of exaggerated polarization (Figure S1). The weak binding energy predicted by PBE can therefore be attributed to electronic polarization in the absence of long-range dispersion contributions. Bader analysis of the geometries optimized with BEEF-vdW (Table S10) showed no significant net charge transfer between the adsorbate and the surface, confirming the absence of covalent character. This interpretation was further supported by the PDOS analysis (Figure 4a), in which the Ni d_*z*_^2^ states of the surface atom were essentially unchanged by adsorption of DBP in the high-coordination geometry. In turn, the PDOS of the carbon p_*z*_ states (Figure 4b) exhibited slight broadening accompanied by a systematic upward shift in energy, indicating redistribution within the π-system.^36^

**Figure 3:**
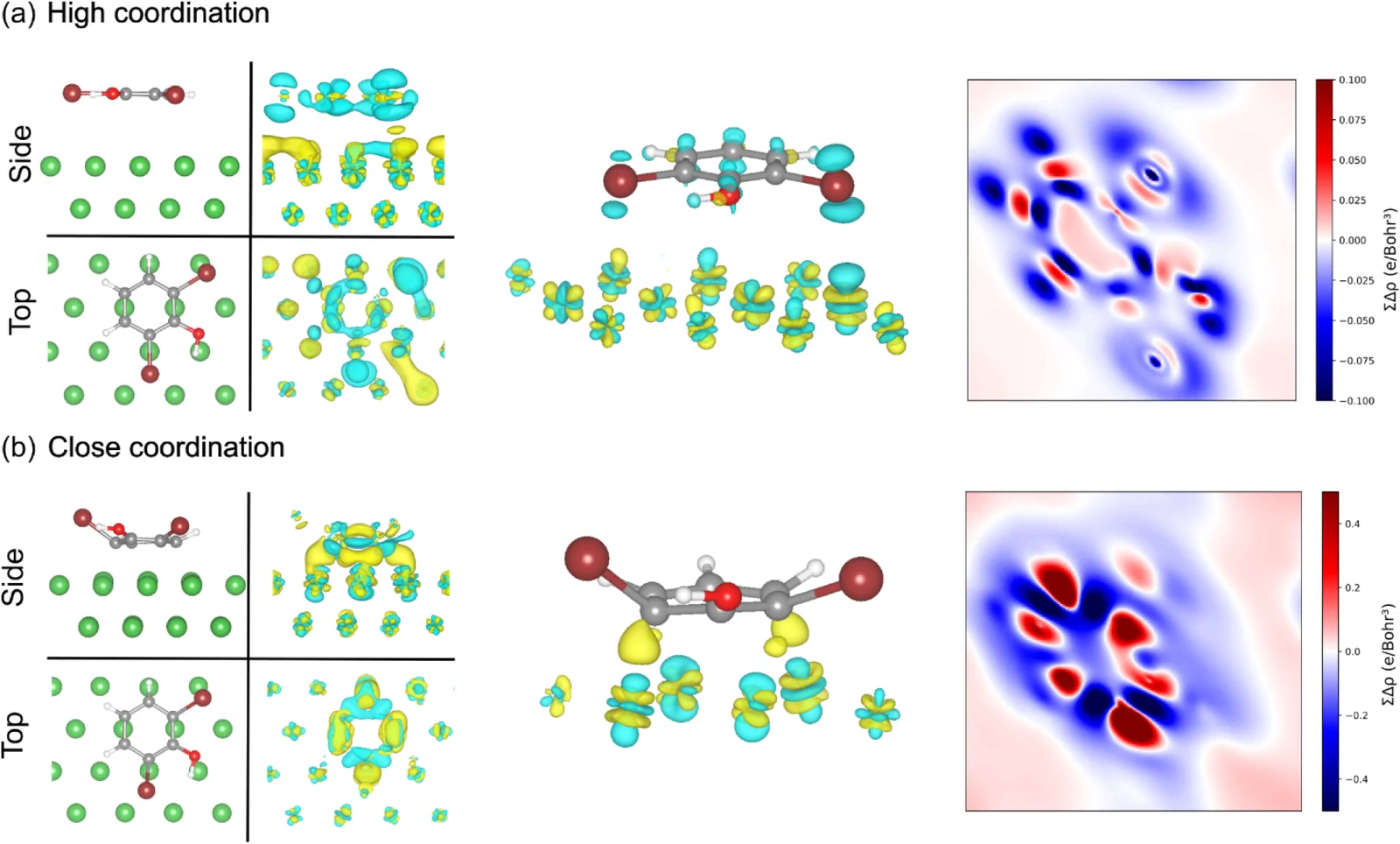
(a) Charge density difference (CDD) plots for 2,6-dibromophenol adsorbed on Ni(111), calculated with the BEEF-vdW functional. (a) High-coordination geometry the physisorption regime, with isosurfaces at 0.0025 e/Å^3^ (left column) and 0.005 e/Å^3^ (middle column). (b) Close-coordination geometry of the chemisorption regime, with isosurfaces at 0.025 e/Å^3^ (left column) and 0.1 e/Å^3^ (middle column). The substantially higher isosurface levels in (b) reflect the larger magnitude of charge redistribution in the chemisorption regime. Right-hand columns show xy-slices of the CDD taken at the average height of the adsorbate. Cyan (blue) regions indicate electron density depletion and yellow (red) regions indicate electron density accumulation. Corresponding CDD plots for 2-bromophenol and phenol are provided in Figure S2.

**Figure 4:**
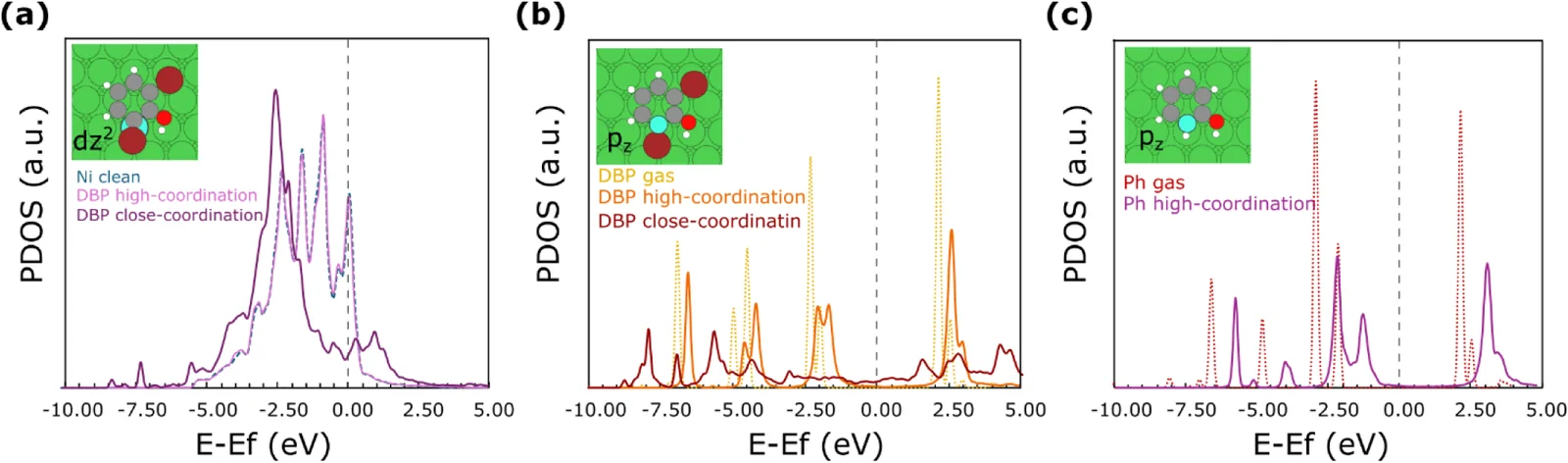
Projected density of states (PDOS) calculated with the BEEF-vdW functional for the high-coordination geometry: (a) Ni d_*z*_^2^ states of the surface nickel atom directly beneath the adsorbed DBP, (b) carbon p*_z_* states of DBP, and (c) carbon p*_z_* states of phenol. The atom on which the projection is performed is highlighted in cyan in the accompanying structural diagrams. Solid lines correspond to the adsorbed system, while dotted lines show the reference state (clean Ni surface for (a), gas-phase molecule for (b) and (c)). Energies are given relative to the Fermi level of the corresponding system.

##### 3.1.1.2 Chemisorption Regime

DBP exhibited weaker binding at shorter surface separations (close-coordination regime) than in the corresponding high-coordination structures, despite the average adsorbate-surface distance (2.17 Å) being within the typical range for chemisorption. Table 1 presents the adsorption energies for the most stable 0° and 30° configurations geometries of DBP on Ni(111), while all studied geometries, together with relevant angular parameters and bond distances, are provided in Tables S1–S3 of the Supplementary Information. Consistent with earlier studies of various aromatic adsorbates,^30,31,37–39^ our simulations identified the bri30 geometry as the most stable close-coordination configuration, with an adsorption energy of −0.53 eV. In contrast, the 0° configuration (bri0) was considerably less stable, exhibiting an adsorption energy of −0.09 eV.

In bri30 geometry shown in Figure 2a, two carbon atoms coordinate directly above nickel atoms (marked red in Figure 1), and are referred to as σ-carbons,^40^ while the remaining four carbons occupy bridge-like π-coordination sites and are referred to as π-carbons^37,41^ (blue in Figure 1). In the most stable configuration, the two σ-carbons bear one C–Br and one C–H substituents, both tilted sharply away from the surface (37° and 26°, respectively). The bromine on the σ-carbon was positioned over an FCC hollow site of the nickel surface. Substituents on the π-carbons remain closer to the surface plane, with tilt angles of 21° for C–Br and 20° for C–OH. Elongation of the C–Br bond at the σ-carbon (2.028 Å) relative to the π-carbon (1.959 Å) and to the physisorption geometry (1.917 Å and 1.926 Å) indicates weakening of the bond. The C–C bonds between σ- and π-carbons (average 1.456 Å) are also elongated relative to those between two π-carbons (average 1.425 Å) and to those in physisorbed DBP (average 1.397 Å). The upward tilting of the substituents and the deformation of the ring together suggest partial rehybridization of the carbon framework from planar sp^2^ toward sp^3^-like character.

The orientation of the functional groups had a notable effect on the adsorption strength at bridge sites. Replacing the OH group with a bromine substituent on a σ-carbon in the bri30 configuration destabilized the adsorption energy by 0.25 eV (Table S1). Configurations in which the bromine substituent was positioned directly above a nickel atom were identified as saddle-point structures: during optimization, the adsorbate migrated laterally away from its initial position.

The CDD analysis of the bri30 adsorption geometry in Figure 3b shows a strong accumulation of electron density between the carbon ring and the nickel surface. Electron density was depleted from both the π-orbitals of the carbon ring and the nickel d-orbitals, indicating mutual donation between adsorbate and surface. Examination of the density-difference map at a higher isosurface level (middle column of Figure 3b) showed that bonding was strongest between nickel and the σ-carbons, with electron density being drawn particularly from the Ni d_*z*_^2^ orbitals. These observations were further supported by the PDOS shown in Figure 4a, where the d_*z*_^2^ states had shifted toward lower energies and exhibited a pronounced resonance below −2.50 eV, implying overlap between the Ni d_*z*_^2^ and the carbon p_*z*_ orbitals of DBP. The chemisorption picture is reinforced by the broadening of the Ni d_*z*_^2^ states near the Fermi level and by the more widespread broadening of the adsorbate p_*z*_ states (Figure 4b), both of which indicate significant hybridization relative to the clean nickel surface and gas-phase DBP. Bader analysis (Table S10) revealed that electron density was transferred from the nickel to the carbon ring, consistent with chemical bonding between the adsorbate and the surface. However, the steric strain induced by the bent substituents appears to offset this stabilization, as reflected by the lower adsorption energy compared with the high-coordination geometry.

At the hollow FCC-sites, the FCC0 and FCC30 geometries exhibited only minor energetic differences, with adsorption energies of −0.31 eV and −0.32 eV, respectively (Table 1). For the HCP sites, slightly stronger binding was obtained (−0.35 eV for HCP0 and −0.37 eV for HCP30), suggesting that the second metal layer directly beneath the HCP site may provide slight additional stabilization through enhanced metal–adsorbate orbital overlap. Hollow 0° configurations (Figure 1), in which six π-carbons coordinate symmetrically with the surrounding nickel atoms, have previously been reported to yield strong adsorption for aromatic compounds.^30,31,37^ For DBP, however, the energy difference between 0° and 30° configurations at the hollow sites was minor. The orientation of bromine and OH substituents had only a small effect on the adsorption energies at hollow sites (Tables S1–S3). As observed for bridge-site adsorption, configurations in which Br or OH was positioned near an atop site were destabilized, leading either to lateral displacement of the aromatic ring or to dissociation of the C–Br bond during optimization. These observations further suggest that such atop-adjacent configurations correspond to saddle-point structures.

vdW-DF predicted little or no binding in the close-coordination regime, consistent with its repulsive character at short adsorbate-surface separations.^35^ The PBE functional, in turn, systematically yielded slightly stronger adsorption energies for close-coordination geometries than those obtained with BEEF-vdW (Table 1), as expected from the known tendency of PBE to overestimate chemisorption energies.^19^ Because PBE substantially underestimates the dispersion contribution that stabilizes the high-coordination geometries, the overestimation of chemisorption energies appears to compensate in part for the absence of nonlocal correlation term. As a result, PBE produces close-coordination adsorption energies that appear broadly comparable to those obtained with BEEF-vdW, which explicitly accounts for dispersion interactions. Adding the D3 correction increased the close-coordination adsorption energies substantially relative to uncorrected PBE, and to a greater extent than in the high-coordination geometries, so that the close-coordination configuration remained the more stable one. This behavior reflects the pairwise nature of the D3 model, in which the dispersion energy is evaluated from atomic positions using coefficients that do not account for screening of the dispersion interaction by the substrate electron density. Such screening reduces the effective dispersion coefficients near a metal surface, and its neglect leads to overbinding that is most pronounced at short adsorbate-surface separations, where the pairwise sum is largest.

Overall, the dispersion-corrected vdW functionals predicted physisorption as the more stable adsorption mode for DBP at the electronic-energy level. To assess structural stability and how vibrational contributions modify this conclusion, vibrational analyses were performed for the most stable physisorbed and chemisorbed bridge-site geometries optimized with the BEEF-vdW functional. Imaginary frequencies were obtained for both configurations even after tightening the convergence criterion from 0.03 to 0.01 eV/Å. These modes originate from artifacts of the partial Hessian approach, and displacing the structures along them relaxed them back to the original geometry, confirming that they do not correspond to structural instabilities. Both the physisorbed and chemisorbed configurations were therefore taken as local minima.

The ZPE correction differed by 0.10 eV between the two configurations, slightly stabilizing the chemisorbed state relative to physisorption. With vdW-DF, the electronic energy gap between the chemisorbed and physisorbed states was substantially larger (∼0.48 eV), leaving the ordering unchanged after ZPE correction. With BEEF-vdW the electronic gap was comparable to the ZPE correction (∼0.1 eV), rendering the two modes close to isoenergetic; the qualitative preference for physisorption therefore rests on the functional with the large gap. This preference is further reinforced by entropy. The physisorbed state retains frustrated translational and rotational character that is largely absent in the more strongly bound chemisorbed state. As these are low-frequency modes, poorly described by the harmonic approximation, they were treated using a hindered-rotor model, which yielded translational and rotational contributions of 0.41 and 0.19 eV (TΔS) for physisorbed DBP at 423.15 K and 2000 Pa. The residual entropy stabilization therefore exceeds the 0.10 eV ZPE difference by a substantial margin, and the physisorbed configuration is expected to remain thermodynamically favored under the relevant reaction conditions.

#### 3.1.2 Bromophenol

Reducing the number of bromine substituents altered the relative stability of the physisorption and chemisorption regimes. For BP, the BEEF-vdW functional predicted nearly equal adsorption strengths at the bri30 site for the high- and close-coordination geometries, with adsorption energies of −0.56 eV and −0.59 eV, respectively (Figure 2b). The corresponding difference at the hollow sites was even smaller. In the most stable close-coordination geometry, the σ-carbon bore the Br substituent while the OH group was located on the π-carbon at bri30 site. Exchanging these substituents destabilized the structure by 0.10 eV, as the Br and OH groups then adopted a less favorable orientation near atop sites. The bri0 geometry for BP was comparatively more stable than for DBP, which can be attributed to the reduced steric strain associated with the single bromine substituent. As with DBP, BP adsorbed slightly more strongly on HCP sites than on FCC sites.

Overall, the adsorption trends in the chemisorption regime largely mirrored those of DBP, with the orientation of the bromine substituent dictating the adsorption strength. The most stable geometries placed the bromine atom above hollow or bridge regions, whereas placement of the bromine above atop sites consistently weakened the binding or resulted in unstable configurations (Tables S4–S6). As observed for DBP, optimization with the vdW-DF functional produced significantly weaker adsorption energies in the close-coordination regime. Notably, PBE functional yielded BP adsorption energies that deviated more strongly from the BEEF-vdW values than was the case for DBP. D3 dispersion correction provided similar trends with DBP, increasing adsorption energies significantly.

#### 3.1.3 Phenol

In contrast to DBP and BP, phenol was less stabilized in the physisorption regime than in the close-coordination geometries. The most stable close-coordination geometry and the corresponding high-coordination geometry are presented in Figure 2c. The weaker physisorption of phenol in the high-coordination geometry, relative to DBP and BP, highlighted the role of bromine polarizability and the enhanced long-range dispersion interactions in the brominated systems. The CDD analysis (Figure S2) comparing the high-coordination geometries of DBP, BP, and phenol revealed that the bromine substituents in DBP and BP promoted a more extensive distribution of electron density, resulting in more extensive polarization. This trend is reflected in the PDOS in Figure 4c, which showed only a slight broadening of the carbon p_*z*_ states of phenol. As observed for DBP, the p_*z*_ states exhibited a systematic shift to higher energies relative to gas-phase phenol, likely originating from Pauli repulsion at the interface and the resulting redistribution of electron density within the π-system.

For close-coordination geometries in the chemisorption regime, phenol exhibited generally stronger adsorption than the brominated phenolic compounds. The trend observed for high-coordination geometries was thus reversed, with adsorption strength increasing as the number of bromine substituents decreased. This observation is consistent with previous studies on substituted anisole.^42^ In agreement with earlier studies of phenol adsorption on Ni(111),^38,43^ Pd(111),^39,44^ and Pt(111)^44^ surfaces, our simulations identified the bri30 geometry as the most stable close-coordination configuration for phenol, with an adsorption energy of −0.57 eV (Figure 2c). All investigated close-coordination orientations are listed in Tables S6–S9. Unlike DBP and BP, experimental adsorption data are available for phenol, with a reported value of −0.75 eV on Ni(111) at a coverage of 0.3 ML and a temperature of 150 K.^45^ The BEEF-vdW functional therefore underestimated the adsorption energy by 0.18 eV, while vdW-DF performed less accurately, underestimating it by 0.45 eV. In contrast, PBE slightly overestimated the adsorption energy by 0.05 eV. This behavior reflects the well-known tendency of PBE to overestimate chemisorption energies on reactive transition metal surfaces.^19^ PBE predicted adsorption energies approximately 0.2 eV stronger than BEEF-vdW for phenol, compared with differences of ∼0.15 eV for BP and ∼0.05 eV for DBP in close-coordination geometries. The more pronounced difference for phenol can be rationalized by its reduced steric strain relative to the brominated adsorbates and by its more electron-rich π-system, since electron withdrawal by bromine substituents in DBP and BP weakens the covalent contribution to chemisorption. Because PBE overestimates covalent bonding contributions, the discrepancy between PBE and BEEF-vdW predictions is largest for the species with the strongest covalent character, namely phenol. Applying the D3 correction to the PBE-optimized close-coordination geometry yielded an adsorption energy of −1.90 eV, overbinding the experimental value of −0.75 eV by 1.15 eV, compared with deviations of −0.18 eV for BEEF-vdW and −0.45 eV for vdW-DF.

Overall, the adsorption studies demonstrated that bromine substitution plays a key role in governing the balance between chemisorption and physisorption on the Ni(111) surface. This behavior is illustrated by the binding energy curves in Figure S3, where DBP exhibits the greatest stabilization at adsorbate-surface separations above 3.25 Å (high-coordination regime), followed by BP. In contrast, phenol shows the weakest stabilization at larger separations but the strongest stabilization at short adsorbate-surface distances (close-coordination regime). Similar behavior has previously been reported for phenol adsorption on Pt(111) with different nonlocal vdW functionals.^46^

### 3.2 Debromination

The debromination of DBP on Ni(111) was examined to provide atomistic insight into the associated reaction energetics. Calculations were performed using the BEEF-vdW functional, as it provided the most balanced description of both the physisorption and chemisorption regimes for the studied phenolic adsorbates.

Only ZPE corrections are included in the reported energies and vibrational entropy contributions are not included, for the following reasons. The vibrational entropy of adsorbates of this type is dominated by low-frequency modes (below ∼100 cm^–1^), whose strongly anharmonic potentials are poorly described by the harmonic approximation; harmonic treatment of these modes yields entropies that differ substantially between near-identical structures without genuine physical significance.^28^ Anharmonic corrections that resolve this (torsional eigenvalue summation and related hindered-rotor and grid-based methods) require explicit mapping of the relevant mode potentials and are tractable only for small systems with a limited number of coupled low-frequency modes,^29^ and are therefore not applicable to a periodic adsorbate on a constrained surface slab, where many such modes are coupled and, under the geometry constraints employed, frequently become imaginary. The size of the studied system limits the tightness of the convergence criteria within reasonable computational cost, and the numerical accuracy achievable with the partial Hessian approach further complicates the reliable evaluation of low-frequency modes. The adopted approach provides an adequate description of the surface-mediated reactions that are the focus of the study. We note that gas-phase species retain their full translational and rotational entropy, which further stabilizes the gas-phase reference states relative to surface-bound intermediates at finite temperature, and that physisorbed species retain some frustrated rotational and torsional character relative to chemisorbed species, providing additional entropic stabilization.

The minimum-energy paths for the reaction steps examined are presented in Figure 5, while transition state energies, rate constants, and reaction energies are summarized in Table 2. As discussed in Section 3.1, DBP preferentially adsorbed in a high-coordination geometry within the physisorption regime. Debromination from this configuration was not feasible, and DBP first had to approach the surface for C–Br bond activation to occur. This reconfiguration step (**1HC** → **1CC**) involved a transition state (**TS1**) with an activation energy of 0.27 eV. Concurrently, the C–Br bond elongated by approximately 0.10 Å (1.924 Å → 2.024 Å), indicating bond weakening, and Bader charge analysis revealed electron density transfer from the nickel surface to the carbon ring (Table S11).

**Figure 5:**
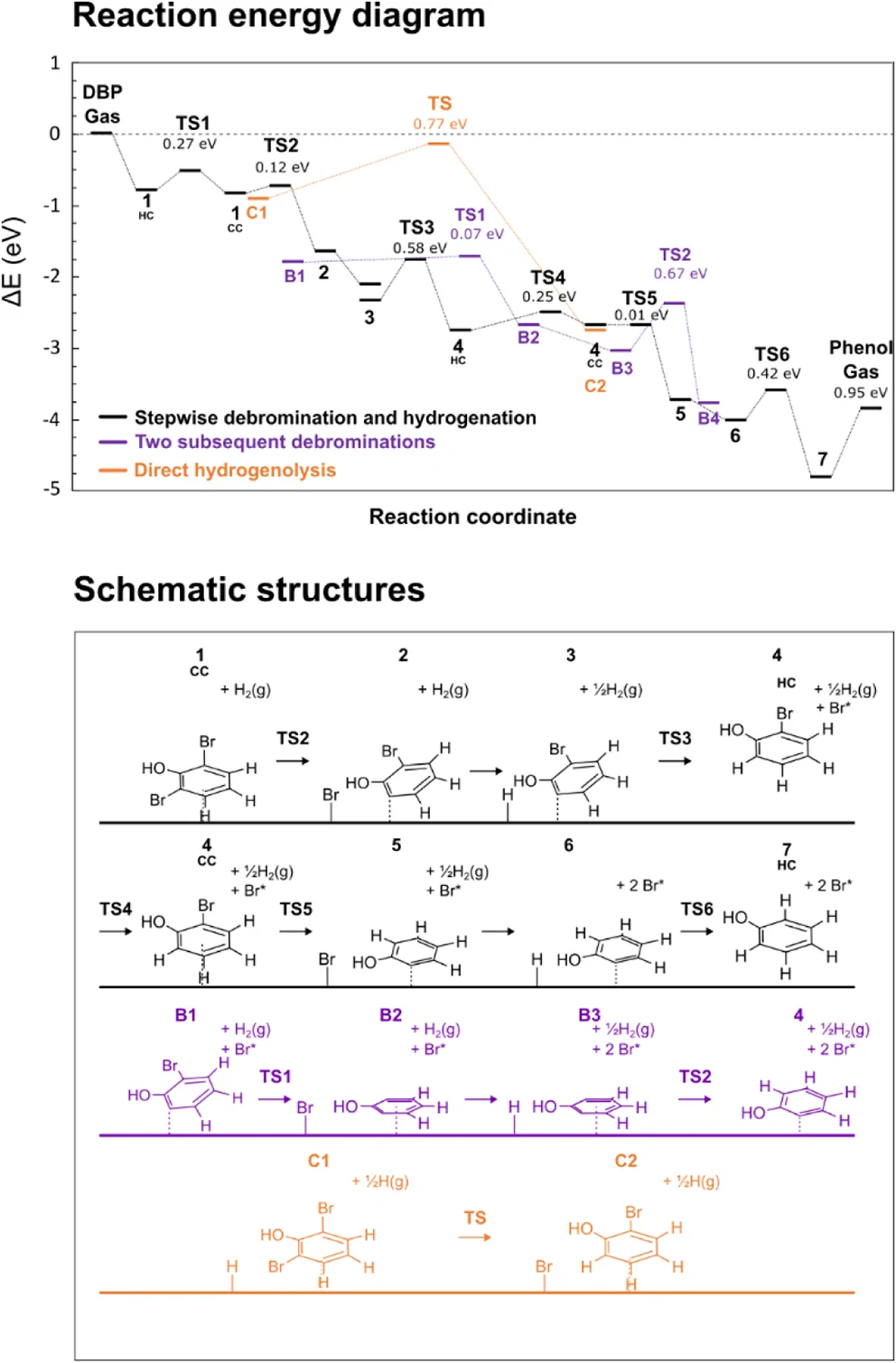
Reaction energy diagram for the three debromination pathways of 2,6-dibromophenol on Ni(111) examined in this work: stepwise debromination-hydrogenation (black), sequential debromination (purple), and direct hydrogenolysis (orange). Reaction energies with ZPE correction are given in eV relative to gas-phase DBP. Activation barriers are indicated above each transition state. Schematic structures of the intermediates along each pathway are shown below the energy diagram. The labels HC and CC denote high-coordination and close-coordination geometries, respectively.

**Table 2:** Activation Energies (TS), Forward and Reverse Rate Constants (k_+_ and k_–_), Reaction Energies (ΔE), and Equilibrium Constants (K_eq_) for the Elementary Steps of the Three Debromination Pathways of 2,6-Dibromophenol on Ni(111), Calculated with the BEEF-vdW functional^a^,^b^

**Reaction information**		**Forward**		**Backward**		**Thermodynamics**	
Reaction	Label	TS (eV)	k_+_ (s^–1^)	TS (eV)	k_–_ (s^–1^)	ΔE (eV)	K_eq_
Physisorption to chemisorption	1HC → 1CC	0.27	5.96 × 10^9^	0.32	1.42 × 10^9^	–0.05	4.21
1st bromine dissociation	**1CC → 2**	0.12	3.42 × 10^11^	0.93	84.0	–0.81	4.07 × 10^9^
1st hydrogenation	**3 → 4HC**	0.58	1.17 × 10^6^	1.00	12.2	–0.42	9.56 × 10^4^
Physisorption to chemisorption	**4HC → 4CC**	0.25	8.23 × 10^9^	0.18	7.24 × 10^10^	0.08	0.11
2nd bromine dissociation	**4CC → 5**	0.01	7.63 × 10^12^	1.06	2.27	–1.05	3.36 × 10^12^
2nd hydrogenation	**6 → 7**	0.42	9.27 × 10^7^	1.21	3.34 × 10^–2^	–0.79	2.78 × 10^9^
2nd bromine dissociation (sequential)	**B1 → B2**	0.07	1.31 × 10^12^	0.96	29.4	–0.89	4.46 × 10^10^
1st hydrogenation	**B3 → B4**	0.67	1.00 × 10^5^	1.39	2.16 × 10^–4^	–0.73	4.64 × 10^8^
Direct hydrogenolysis	**C1 → C2**	0.77	6.58 × 10^3^	2.60	1.03 × 10^–18^	–1.83	6.36 × 10^21^

a Values have been calculated from electronic energies including a ZPE correction.

b Rate-constants were computed from the Eyring equation at 150 °C, chosen based on the experimental conditions of refs 13 and 14, with the activation entropy assumed to be negligible (see Methods). The reported values are intended for relative comparison among the elementary steps rather than as absolute predictions of catalytic turnover.

The debromination step (**1CC** → **2)** proceeded from the close-coordination geometry and was highly exothermic, with a reaction energy of −0.81 eV and a relatively low activation barrier of 0.12 eV (**TS2**). The minimum-energy reaction path obtained from the NEB calculation is presented in Figure 6a. Bader analysis (Figure 6a and Table S12) reveals a redistribution of electron density during the metal mediated C–Br cleavage, with the dissociating bromine gaining electron density, accompanied by a corresponding loss of electron density from Ni(111) surface. The adsorption of bromine on Ni(111) was highly stabilizing, with an adsorption energy of −1.45 eV when referenced to HBr (−1.84 eV referenced to Br_2_), thereby providing a strong thermodynamic driving force for debromination. The reverse reaction was associated with a high activation barrier of 0.93 eV, rendering the debromination step practically irreversible under the conditions studied. This was reflected by the forward rate constant (k_+_) exceeding the reverse rate constant (k_–_) by ten orders of magnitude (Table 2). In the gas-phase, a DFT study reported the C–Br bond dissociation energy in 2,6-dibromophenol to be approximately 3.56 eV^15^, while the experimental value for bromobenzene is 3.62 eV.^47^ Surface studies of the successive debromination of hexabromobenzene on Ag(111) reported the first debromination step to be exothermic by 0.62 eV with an activation barrier of 0.26 eV.^48^ While the activation barrier is comparable to that obtained in the present study, the debromination on Ni(111) was notably more exothermic, which can be attributed to the stronger adsorption and stabilization of bromine on nickel relative to silver.

**Figure 6:**
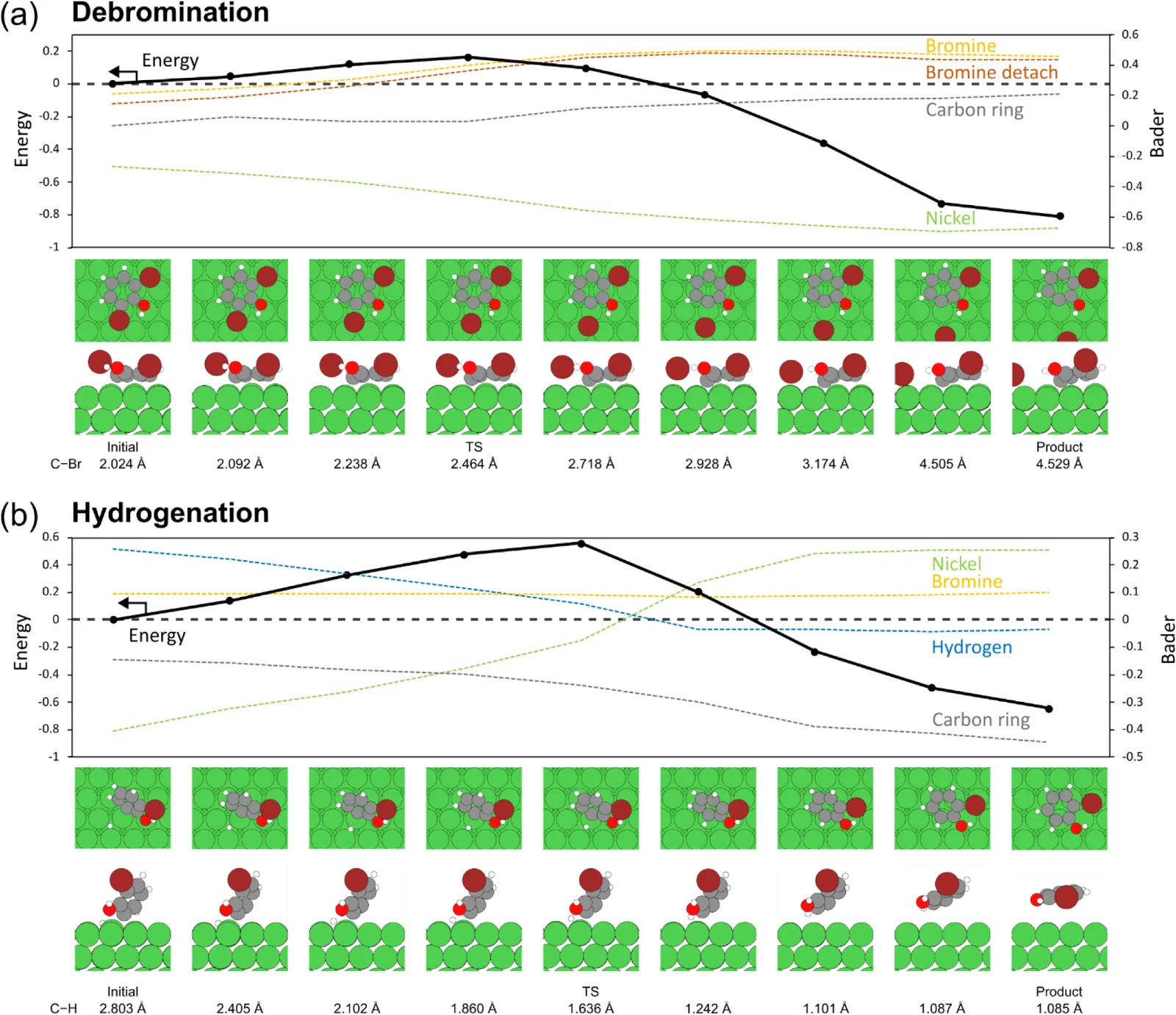
Minimum-energy paths (presented without ZPE correction) and Bader charge evolution for (a) the first debromination step (**1CC** → **2**) and (b) the subsequent hydrogenation step (**3 → 4HC**) of 2,6-dibromophenol on Ni(111), calculated with the NEB method and the BEEF-vdW functional. Energies along the reaction coordinate are shown by the black solid line (left axis, eV). Bader charges of the indicated atoms or atomic groups are shown by colored dashed lines (right axis, e). Structural snapshots along each path are presented below the energy curves, with the relevant C–Br or C–H bond distance indicated.

Following the initial debromination, the reaction may proceed through hydrogenation (**3 → 4HC**), for which the minimum-energy pathway is shown in Figure 6b. Although hydroxyl orientation did not influence the barrier height, configurations with the OH group directed away from the surface were thermodynamically more stable. The hydrogenation step (**3 → 4HC**) was exothermic by 0.42 eV and exhibited an activation barrier of 0.58 eV. Previously, hydrogenation of the phenyl radical to benzene on Ni(111) was reported to involve a notably lower barrier of 0.30 eV with the PBE functional.^49^ This difference likely originates from the inability of PBE to account for vdW interactions.

The preferred hydrogenation pathway involved an upright configuration of the bromoaryl intermediate on the surface, as shown in Figure 6b. Bader analysis indicated a redistribution of electron density during the surface-mediated hydrogenation, with electron density transferred from the carbon ring and the approaching hydrogen atom to the nickel surface. Along the energetically most favorable pathway, the resulting BP remained adsorbed in a high-coordination geometry. A hydrogenation pathway leading to BP adsorption in a close-coordination geometry was also identified; this route, however, was associated with a slightly higher activation barrier (∼0.2 eV). In addition, the possibility of hydrogen transfer from the OH group to the debrominated carbon atom, yielding an adsorbed phenoxide-like species with an added H at the former Br position, was examined. While restoration of aromaticity rendered this scheme energetically favorable (ΔE = −1.16 eV), the high activation barrier of 1.12 eV indicated that this process was kinetically unlikely. The various hydrogenation pathways examined are summarized in Figure S4. On the basis of these results, the hydrogenation step was kinetically more demanding than the preceding debromination step.

After completion of the first debromination-hydrogenation cycle, the process proceeded analogously with BP to yield phenol. The debromination of the second bromine (**4 → 5**) was practically barrierless (activation barrier 0.01 eV) and highly exothermic, with a reaction energy of −1.05 eV. Cleavage of the second C–Br bond was therefore even more accessible than the initial debromination step. The subsequent hydrogenation step to form phenol (**6 → 7**) was similarly exothermic by 0.79 eV and exhibited an activation barrier of 0.42 eV, and thus slightly lower compared to the first hydrogenation step. Desorption of the formed phenol was endothermic by 0.95 eV without considering the entropic stabilization or possible solvation effects. A previous study on Ag(111) reported that the successive debromination steps of hexabromobenzene displayed substantial variability in activation barriers, ranging from 0.06 to 0.76 eV.^48^ That study did not consider hydrogenation of the ring.

As an alternative, following the first debromination, the reaction could proceed directly through a second debromination step (**B1 → B2**). Along this route, the second bromine removal was more accessible than the first C–Br cleavage, with a reaction energy of −0.89 eV and a low activation barrier of 0.07 eV. The fully debrominated phenolic intermediate bound closely to the surface through the unsubstituted carbon atoms, resulting in pronounced distortion of the aromatic ring into a boat-like geometry, indicating a loss of aromaticity. The subsequent hydrogenation (**B3 → B4**) was exothermic by 0.73 eV but involved a higher activation barrier of 0.67 eV than the hydrogenation step following the first debromination. This increase in the activation barrier can be ascribed to stronger Ni–C bonding between the debrominated carbon atoms and the nickel surface.

As a third option, direct hydrogenolysis, in which debromination and hydrogenation occur in a single elementary step (**C1 → C2**), was investigated. This pathway is presented in Figure 5 and Figure S5. Bader analysis indicated that the nickel surface gained electron density from the approaching hydrogen atom, and that beyond the TS both hydrogen and the surface donated electron density to the detaching bromine atom (Table S14). This reaction was highly exothermic, with a reaction energy of −1.83 eV, but was associated with an activation energy of 0.77 eV. This activation barrier was notably higher than those of the individual stepwise debromination and hydrogenation steps. For comparison, gas-phase DFT simulations reported an activation barrier of 0.36 eV for the reaction between TBBPA and atomic hydrogen leading to bromine removal.^16^ This value is referenced to atomic hydrogen, however, which is highly reactive and not directly comparable to surface-mediated hydrogenation processes.

On the basis of these results, the stepwise debromination-hydrogenation route was identified as the most favorable reaction pathway, compared with both direct hydrogenolysis and sequential debromination prior to hydrogenation. This was primarily because the initial debromination step involved a low activation barrier while providing significant stabilization through the adsorbed bromine. Although debromination itself is energetically favorable, the resulting strong binding of bromine leads to progressive blockage of active sites, thereby deactivating the catalyst. Even neglecting activation barriers, the desorption of surface-bound bromine as either HBr or Br_2_ is energetically demanding. The desorption of Br* with a surface hydrogen atom to form HBr was endothermic by 1.70 eV, while desorption of two adjacent Br* atoms to form Br_2_ was endothermic by 1.82 eV per bromine. Both removal channels are therefore thermodynamically considerably uphill compared with the debromination chemistry, supporting the conclusion that Br* accumulates on the surface rather than desorbing. Overall, these findings establish nickel as an active, earth-abundant catalyst for debromination, while also identifying the strong adsorption of bromine as the principal limitation to sustained catalytic activity. One practical mitigation strategy against bromine-induced halogen poisoning is the combined use of protic solvents and alkaline halogen scavengers.^13^

## 4 Conclusions

The adsorption and debromination of 2,6-dibromophenol on Ni(111) reveal a surface chemistry shaped by competing dispersion and covalent contributions, with the balance set by the degree of bromine substitution. While phenol preferentially chemisorbs at ∼2.2 Å from the surface, 2,6-dibromophenol is more stable in a physisorbed configuration at ∼3.5 Å, and 2-bromophenol sits near the crossover. This reversal originates from the enhanced polarizability of the C–Br bonds, which strengthens long-range dispersion interactions sufficiently to outweigh the covalent stabilization available in close-coordination geometries. Halogen substitution can therefore shift aromatic adsorbates away from the strongly bound chemisorbed states characteristic of their unsubstituted analogues, a consideration relevant to any catalytic dehalogenation of polyhalogenated aromatics on transition-metal surfaces. Capturing this effect places a stringent requirement on the choice of functional: BEEF-vdW and vdW-DF reproduce the bromine-induced stabilization, while PBE misses it entirely. Adding a pairwise D3 correction to PBE does not resolve this: it produces strong overbinding, particularly in the close-coordination geometries, and overestimates the experimental phenol adsorption energy by more than 1 eV. This reflects the absence of density-dependent screening in pairwise dispersion models. DFT studies of halogenated aromatic adsorption must therefore explicitly account for non-local correlation.

The mechanistic analysis identifies a stepwise debromination-hydrogenation sequence as the kinetically preferred pathway, with ring hydrogenation rather than C–Br cleavage as the kinetically demanding step (0.58 eV barrier versus 0.12 eV for the initial debromination). Direct hydrogenolysis is disfavored (0.77 eV barrier), and sequential removal of both bromines prior to hydrogenation is impeded by the pronounced distortion of the dibrominated intermediate, which sacrifices aromaticity. The dominant practical limitation, however, is not the activation of the C–Br bond but the fate of the bromine atom after cleavage. The strong adsorption of Br on Ni(111) makes possible desorption channels thermodynamically unfavorable: HBr formation is endothermic by 1.70 eV, while Br_2_ formation is endothermic by 3.64 eV (1.82 eV per Br). Surface-bound bromine cannot therefore leave the catalyst spontaneously. This poisoning is an inherent consequence of the same Br–Ni interactions that drive the high thermodynamic favorability of debromination. Halogen scavengers or surface regeneration are therefore not optional refinements but essential components of any practical nickel-catalyzed debromination process, particularly for industrial applications such as the debromination of BFR containing pyrolysis oils. Future work should explore these strategies experimentally, as well as investigate alternative nickel-based materials with reduced susceptibility to halogen poisoning.

These conclusions rest on a deliberately simplified model. The pristine Ni(111) terrace neglects the undercoordinated edge and corner sites that often dominate the reactivity of supported nanoparticles, the catalyst-support interactions present in Ni/Al_2_O_3_ systems, and the protic-solvent environment of the experimental debromination. Coverage effects, particularly the influence of accumulating surface bromine on subsequent reaction steps, also fall outside the scope of the present calculations and constitute the most immediate priority for future work given the central role of poisoning identified here. Within these caveats, the present results provide an atomistic foundation for understanding the adsorption and debromination of brominated phenolic compounds on nickel, and a starting point for the mechanistic interpretation of tetrabromobisphenol A dehalogenation and related polybrominated systems on earth-abundant catalysts.

## Supplementary Material



## Notes

Tabulated structural parameters for all investigated chemisorption geometries of 2,6-dibromophenol (DBP), 2-bromophenol (BP), and phenol (Ph) on Ni(111) surface. Tabulated Bader charge values for various reaction steps of the debromination pathway. Additional charge-density difference maps for physisorbed adsorbates on Ni(111). Binding-energy curves for DBP, BP, and Ph on Ni(111). Figure of the investigated hydrogenation pathways for the surface-mediated hydrogenation of the debrominated intermediate on Ni(111). Figure of the minimum energy path and Bader charge analysis for the direct hydrogenolysis reaction route on Ni(111) (PDF)
