# Supplementary material for: Adsorption and Catalytic Debromination of 2,6-Dibromophenol on Ni(111): A First-Principles Mechanistic Study
Source: J Phys Chem C Nanomater Interfaces. 2026 Sep 9;130(38):13138–49. doi: 10.1021/acs.jpcc.6c03510 (PMC13618219; doi:10.1021/acs.jpcc.6c03510)
Supplement: Supplementary file 1 [file jp6c03510_si_001.pdf]

Supporting Information

Adsorption and Catalytic Debromination of 2,6−Dibromophenol on Ni(111): A First-Principles Mechanistic Study

Paavo Auvinen*, Mikko Linnolahti, Ville Nissinen, Mika Suvanto, Janne Jänis and Jarkko J. Saarinen
Department of Chemistry and Sustainable Technology, University of Eastern Finland, P.O. Box 111, FI-80101 Joensuu, Finland
*Corresponding author

Table S1 Studied close-coordination geometries for 2,6-dibromophenol on bridge site calculated with BEEF-vdW.

Name	E_{ads} (eV)	C-C Ni-Ni angle (°)	Angles (°)	C-C bonds (Å)	C-Br bonds (Å)	Avg distance from slab (Å)	Optimized geometry
Bri30 - 1	−0.53	31	C3−H1: 16<br>C4−H2: 16<br>C5−H3: 26<br>C6−Br1: 21<br>C2−Br2: 37<br>C1−OH: 20	C1−C2: 1.470<br>C2−C3: 1.467<br>C3−C4: 1.423<br>C4−C5: 1.443<br>C5−C6:1.454<br>C6−C1:1.426	Br1: 1.959<br>Br2: 2.028	2.165	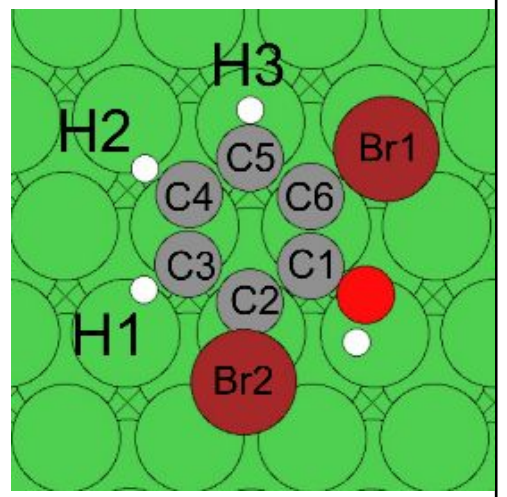
Bri30 – 2	−0.28	30	H1: 16<br>H2: 25<br>H3: 17<br>Br1: 22<br>Br:2 24<br>OH: 34	C1−C2: 1.477<br>C2−C3: 1.435<br>C3−C4: 1.440<br>C4−C5: 1.442<br>C5−C6:1.432<br>C6−C1:1.465	Br1: 1.962<br>Br2: 1.978	2.161	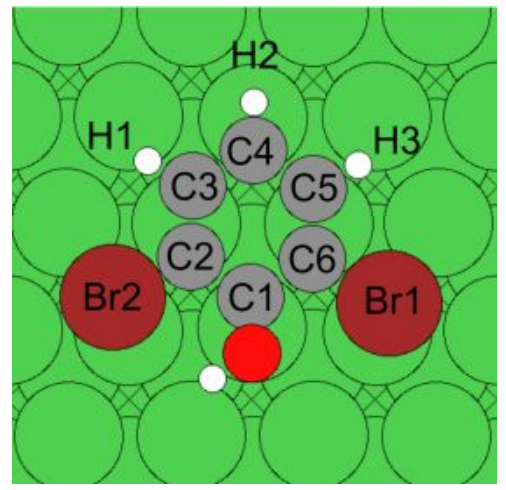
Bri0-1	Saddle point<br>Migrates	-	-	-	-	-	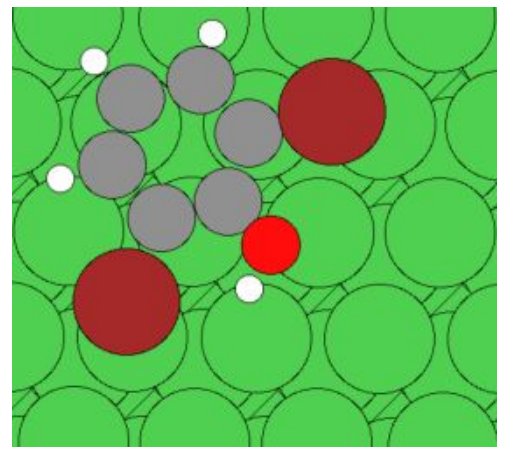

Bri0-2	−0.09	0	H1: 20<br>H2: 18<br>H3: 20<br>Br:1 29<br>Br2: 34<br>OH: 34	C1−C2: 1.476<br>C2−C3: 1.445<br>C3−C4: 1.434<br>C4−C5: 1.436<br>C5−C6:1.437<br>C6−C1: 1.468	Br1: 1.969<br>Br2: 2.000	2.131	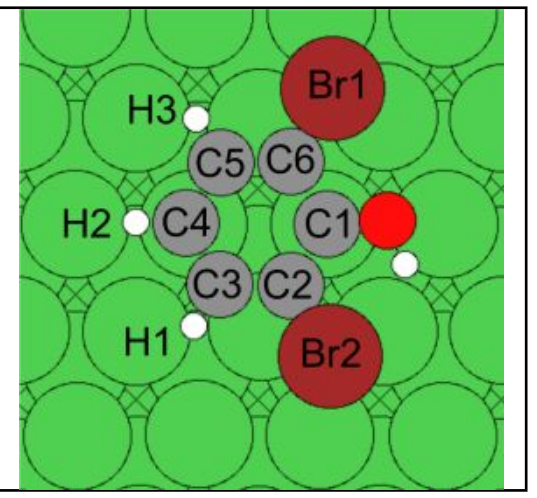

Table S2 Studied close-coordination geometries for 2,6-dibromophenol on FCC hollow site calculated with BEEF-vdW.

Name	E_{ads} (eV)	C-C Ni-Ni angle (°)	Angles (°)	C-C bonds (Å)	C-Br bonds (Å)	Avg Distance from slab (Å)	Optimized geometry
FCC30-1	Saddle point Migrates	-	-	-	-	-	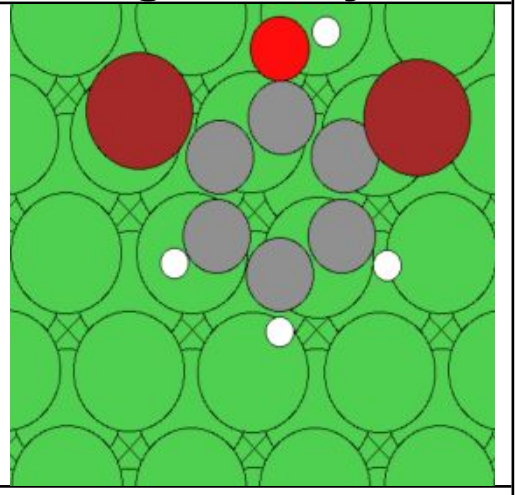
FCC30-2	−0.30	28	H1: 14<br>H2: 18<br>H3: 15<br>Br1: 25<br>Br2: 28<br>OH: 18	C1−C2: 1.441<br>C2−C3: 1.447<br>C3−C4: 1.429<br>C4−C5: 1.433<br>C5−C6:1.441<br>C6−C1: 1.435	Br1: 1.958<br>Br2: 1.985	2.221	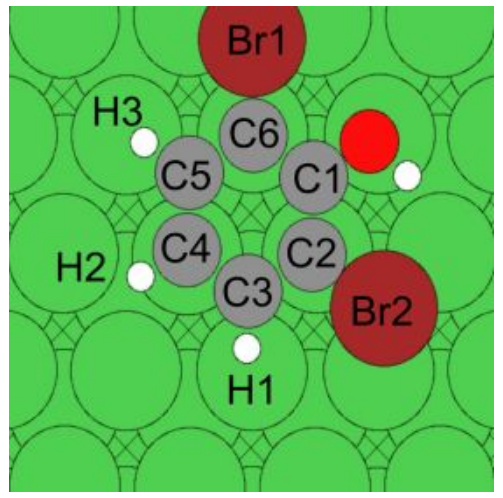
FCC30-3	Saddle point Dissociates	-	-	-	-	-	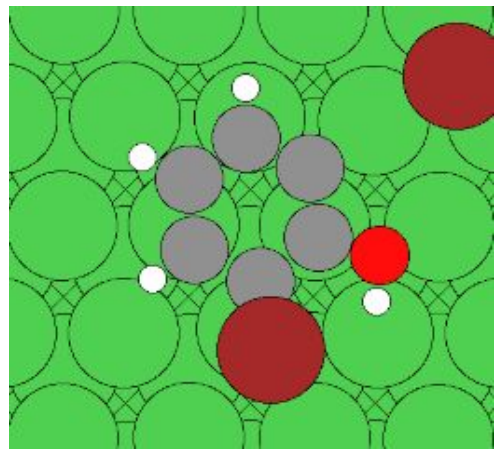
FCC30-4	−0.32	32	H1: 14<br>H2: 18<br>H3: 15<br>Br1: 25<br>Br2: 28<br>OH: 17	C1−C2: 1.445<br>C2−C3: 1.450<br>C3−C4: 1.426<br>C4−C5: 1.434<br>C5−C6:1.444<br>C6−C1:1.431	Br1: 1.956<br>Br2: 1.992	2.207	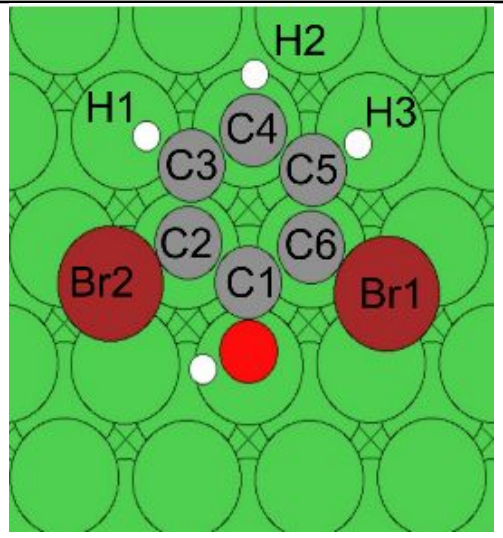

FCC0-1	−0.31	0	H1: 18<br>H2: 17<br>H3: 18<br>Br1: 24<br>Br2: 26<br>OH: 22	C1−C2: 1.466<br>C2−C3: 1.434<br>C3−C4: 1.442<br>C4−C5: 1.421<br>C5−C6:1.455<br>C6−C1:1.431	Br1: 1960<br>Br2: 1975	2.200	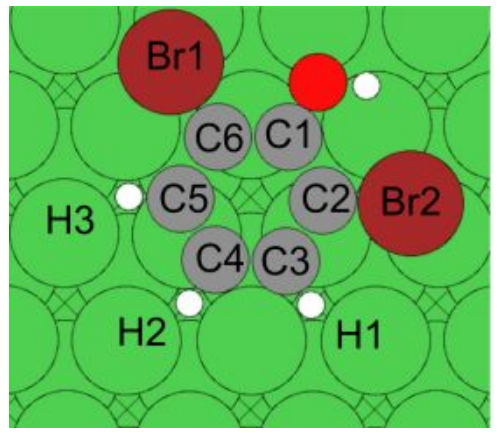
FCC0-2	−0.31	1	H1: 18<br>H2: 17<br>H3: 18<br>Br1: 23<br>B2: 28<br>OH: 22	C1−C2: 1.436<br>C2−C3: 1.457<br>C3−C4: 1.421<br>C4−C5: 1.443<br>C5−C6:1.432<br>C6−C1:1.459	Br1: 1.961<br>Br2: 1.979	2.182	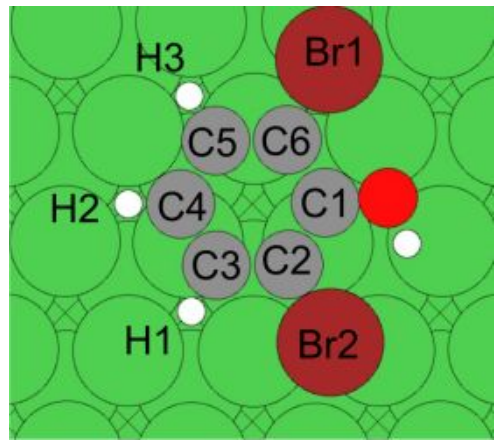
FCC0-3	−0.31	1	H1: 19<br>H2: 17<br>H3: 17<br>Br1: 24<br>Br2: 26<br>OH: 22	C1−C2: 1.465<br>C2−C3: 1.436<br>C3−C4: 1.443<br>C4−C5: 1.419<br>C5−C6:1.455<br>C6−C1:1.431	Br1: 1.961<br>Br2: 1.975	2.188	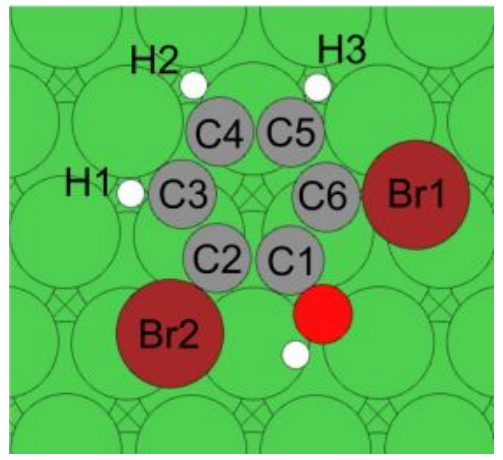

Table S3 Studied close-coordination geometries for 2,6-dibromophenol on HCP hollow site calculated with BEEF-vdW.

Name	E_{ads} (eV)	C-C Ni-Ni angle (°)	Angles (°)	C-C bonds (Å)	C-Br bonds (Å)	Avg Distance from slab (Å)	Optimized geometry
HCP30-1	−0.37	30	C-H1: 14<br>C-H2: 17<br>C-H3: 15<br>Br1: 25<br>Br2: 28<br>OH: 18	C1−C2: 1.445<br>C2−C3: 1.449<br>C3−C4: 1.428<br>C4−C5: 1.431<br>C5−C6:1.446<br>C6−C1:1.431	Br1: 1.957<br>Br2: 1.988	2.188	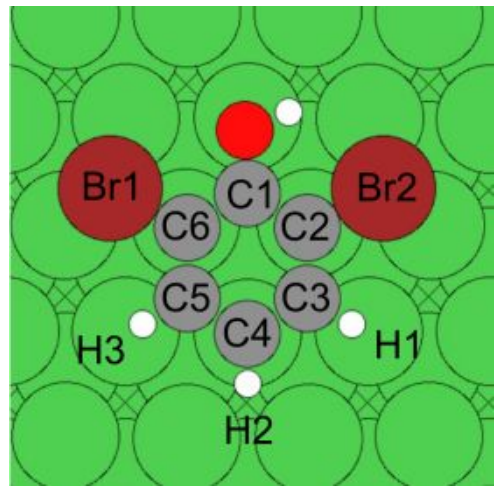
HCP30-2	Saddle point<br>Migrates	-	-	-	-	-	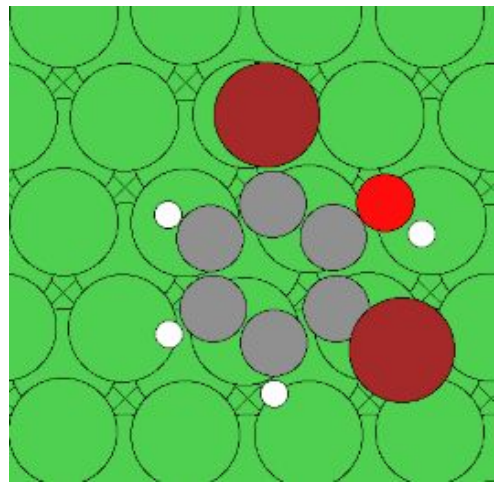
HCP30-3	−0.37	31	H1: 15<br>H2: 18<br>H3: 15<br>Br1: 25<br>Br2: 28<br>OH: 18	C1−C2: 1.445<br>C2−C3: 1.449<br>C3−C4: 1.428<br>C4−C5: 1.431<br>C5−C6:1.446<br>C6−C1:1.431	Br1: 1.962<br>Br2: 1.980	2.186	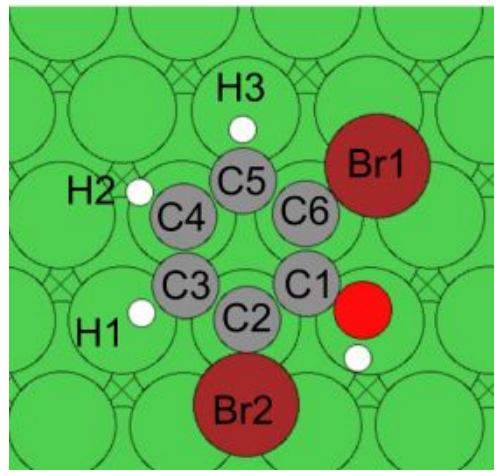

HCP-30-4	Saddle point<br>Dissociates	-	-	-	-	-	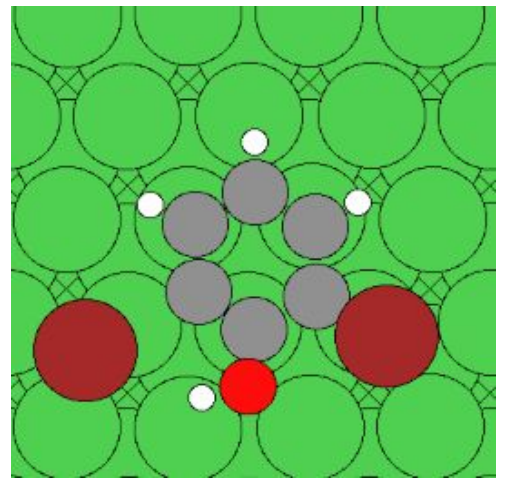
HCP0-1	−0.34	2	H1: 17<br>H2: 17<br>H3: 18<br>Br1: 24<br>Br2: 28<br>OH: 22	C1−C2: 1.438<br>C2−C3: 1.458<br>C3−C4: 1.420<br>C4−C5: 1.443<br>C5−C6:1.433<br>C6−C1:1.458	Br1: 1.961<br>Br2: 1.982	2.213	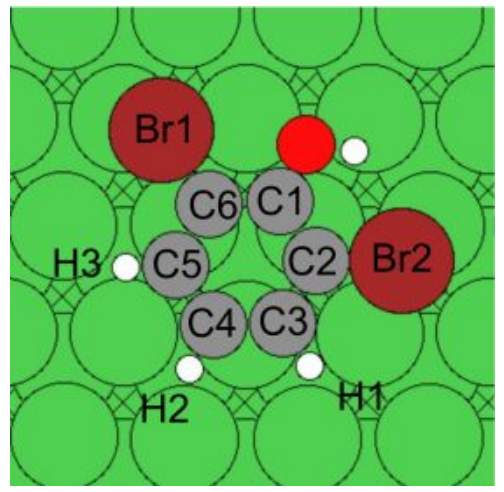
HCP0-2	−0.35	1	H1: 18<br>H2: 17<br>H3: 17<br>Br1: 24<br>Br2: 26<br>OH: 22	C1−C2: 1.464<br>C2−C3: 1.435<br>C3−C4: 1.443<br>C4−C5: 1.420<br>C5−C6:1.456<br>C6−C1:1.432	Br1: 1.961<br>Br2: 1.975	2.196	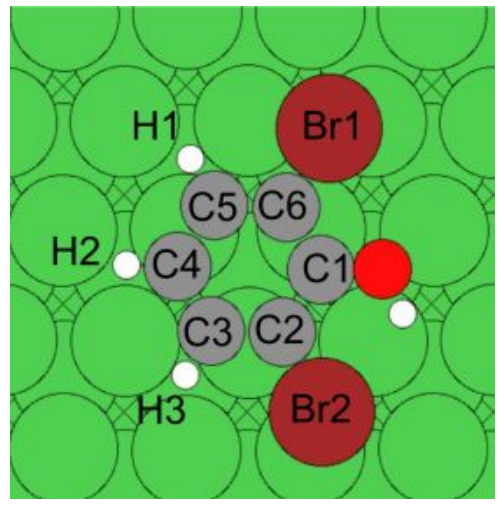
HCP0-3	−0.33	31	H1: 17<br>H2: 17<br>H3: 18<br>Br1: 23<br>Br2: 28<br>OH: 21	C1−C2: 1.435<br>C2−C3: 1.457<br>C3−C4: 1.420<br>C4−C5: 1.443<br>C5−C6:1.433<br>C6−C1:1.457	Br1: 1.962<br>Br2: 1.979	2.179	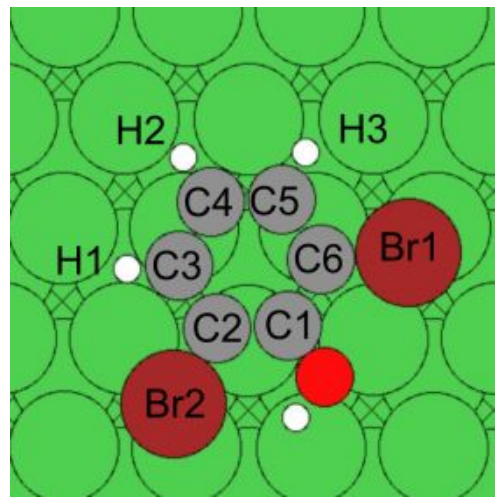

Table S4 Studied close-coordination geometries for 2-bromophenol on bridge site calculated with BEEF-vdW.

Name	E_{ads} (eV)	C-C Ni-Ni angle (°)	Angles (°)	C-C bonds (Å)	C-Br bonds (Å)	Avg Distance from slab (Å)	Optimized geometry
Bri30-1	−0.59	32	H1: 17<br>H2: 24<br>H3: 15<br>H4: 16<br>Br: 33<br>OH: 20	C1−C2: 1.427<br>C2−C3: 1.447<br>C3−C4: 1.442<br>C4−C5: 1.4326<br>C5−C6: 1.462<br>C6−C1: 1.462	1.990	2.16	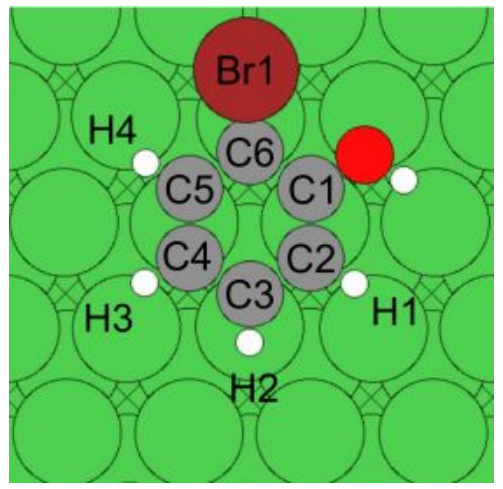
Bri30-2	−0.51	31	H1: 26<br>H2: 14<br>H3: 15<br>H4: 25<br>Br: 22<br>OH: 20	C1−C2: 1.445<br>C2−C3: 1.450<br>C3−C4: 1.424<br>C4−C5: 1.444<br>C5−C6:1.455<br>C6−C1:1.427	1.954	2.183	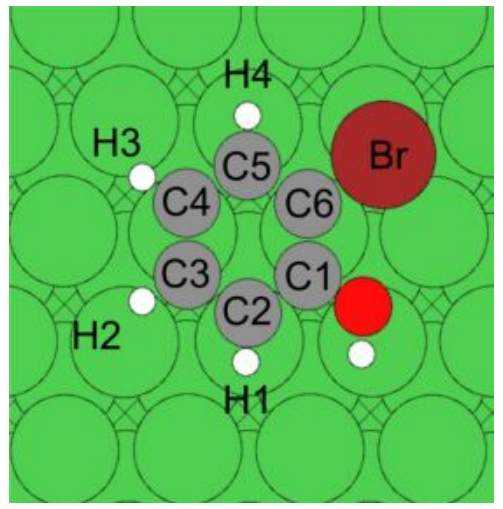
Bri30-3	−0.41	31	H1: 17<br>H2: 15<br>H33: 24<br>H4:17<br>Br: 22<br>OH: 33	C1−C2: 1.464<br>C2−C3: 1.430<br>C3−C4: 1.440<br>C4−C5: 1.444<br>C5−C6:1.431<br>C6−C1:1.456	1.952	2.158	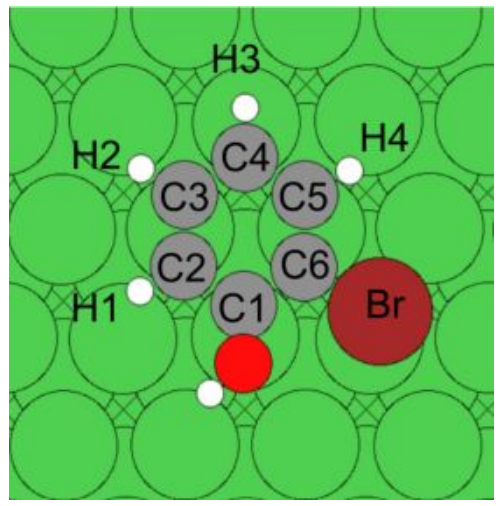
Bri0-1	−0.22	1	H1: 21<br>H2: 19<br>H3: 18<br>H4: 20<br>Br: 29<br>OH: 24	C1−C2: 1.454<br>C2−C3: 1.428<br>C3−C4: 1.437<br>C4−C5: 1.439<br>C5−C6:1.440<br>C6−C1:1.458	1.964	2.149	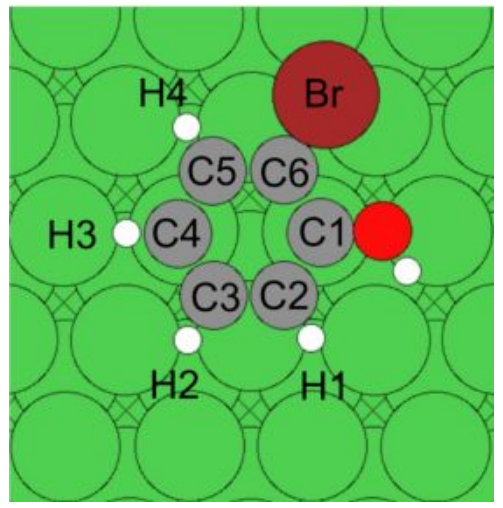

Bri0-2	−0.30	2	H1: 20<br>H2: 17<br>H3: 17<br>H4: 20<br>Br: 21<br>OH: 21	C1−C2: 1.426<br>C2−C3: 1.448<br>C3−C4: 1.437<br>C4−C5: 1.425<br>C5−C6: 1.454<br>C6−C1: 1.445	1.965	2.176	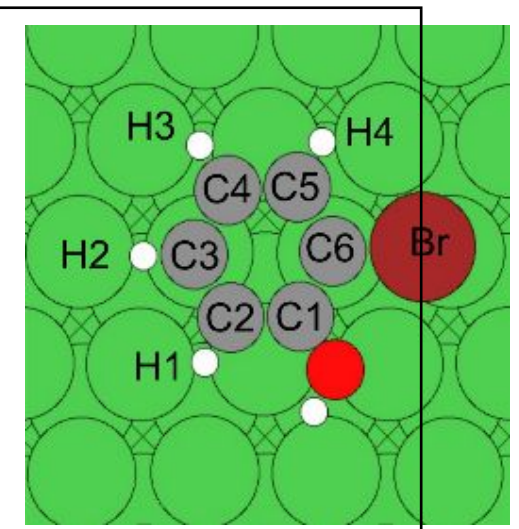
Bri0-3	Saddle point<br>Migrates	-	-	-	-	-	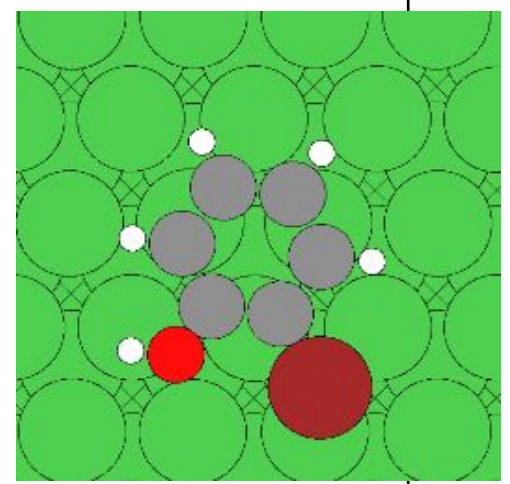

Table S5 Studied close-coordination geometries for 2-bromophenol on FCC hollow site calculated with BEEF-vdW.

Name	E_{ads} (eV)	C-C Ni-Ni angle (°)	Angles (°)	C-C bonds (Å)	C-Br bonds (Å)	Avg Distance from slab (Å)	Optimized geometry
FCC30-1	Saddle point<br>Migrates	-	-	-	-	-	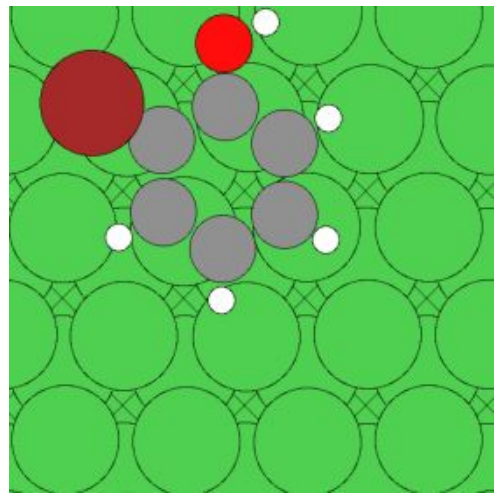
FCC30-2	−0.37	29	H1: 18<br>H2: 12<br>H3: 16<br>H4: 13<br>Br: 25<br>OH: 16	C1−C2: 1.426<br>C2−C3: 1.434<br>C3−C4: 1.426<br>C4−C5: 1.434<br>C5−C6: 1.440<br>C6−C1: 1.434	1.953	2.244	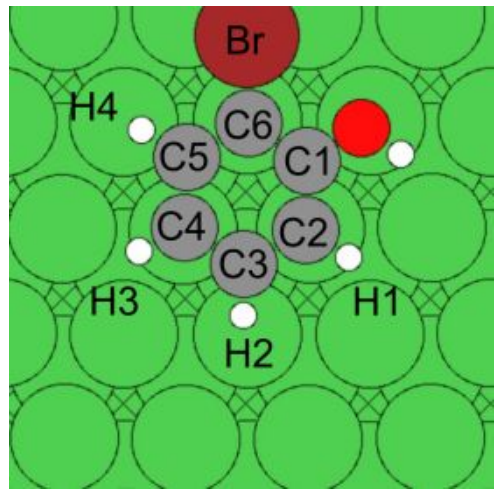
FCC30-3	−0.24	31	H1: 17<br>H2: 17<br>H3: 13<br>H4: 19<br>Br: 21<br>OH: 21	C1−C2: 1.444<br>C2−C3: 1.440<br>C3−C4: 1.427<br>C4−C5: 1.433<br>C5−C6: 1.432<br>C6−C1: 1.425	1.929	2.215	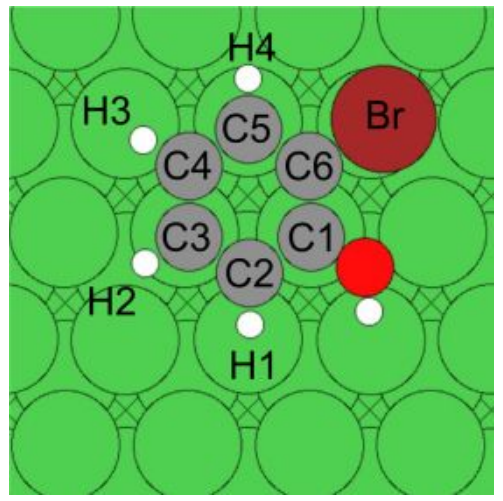
FCC30-4	−0.38	30	H1: 18<br>H2: 13<br>H3: 16<br>H4: 13<br>Br:25<br>OH:16	C1−C2: 1.428<br>C2−C3: 1.435<br>C3−C4: 1.424<br>C4−C5: 1.432<br>C5−C6: 1.443<br>C6−C1: 1.432	1.953	2.230	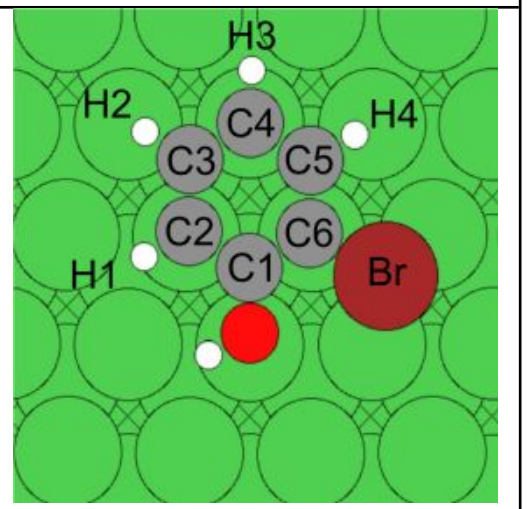

FCC30-5	−0.24	29	H1: 15<br>H2: 18<br>H3: 12<br>H4: 18<br>Br: 23<br>OH: 21	C1−C2: 1.446<br>C2−C3: 1.437<br>C3−C4: 1.431<br>C4−C5: 1.433<br>C5−C6: 1.428<br>C6−C1: 1.425	1.925	2.212	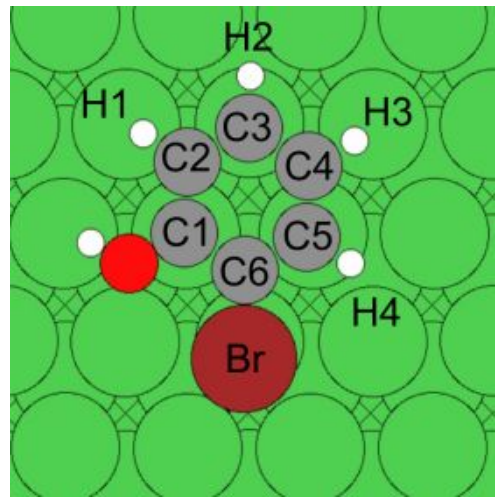
FCC30-6	−0.38	29	H1: 18<br>H2: 12<br>H3: 16<br>H4: 13<br>Br: 24<br>OH 16	C1−C2: 1.424<br>C2−C3: 1.436<br>C3−C4: 1.426<br>C4−C5: 1.431<br>C5−C6: 1.440<br>C6−C1: 1.436	1.956	2.242	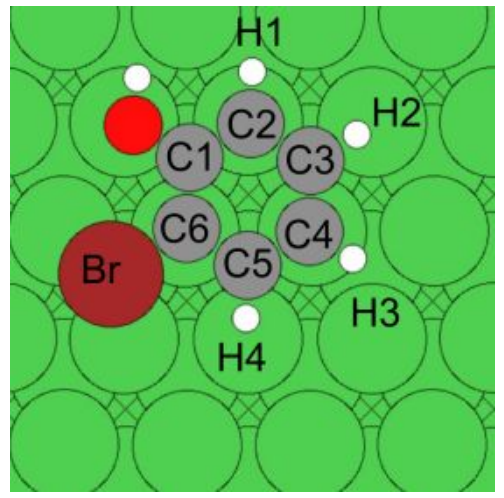
FCC0-1	−0.43	0	H1: 19<br>H2: 17<br>H3: 17<br>H4: 17<br>Br: 24<br>OH: 22	C1−C2: 1.453<br>C2−C3: 1.425<br>C3−C4: 1.443<br>C4−C5: 1.423<br>C5−C6: 1.455<br>C6−C1: 1.426	1.952	2.204	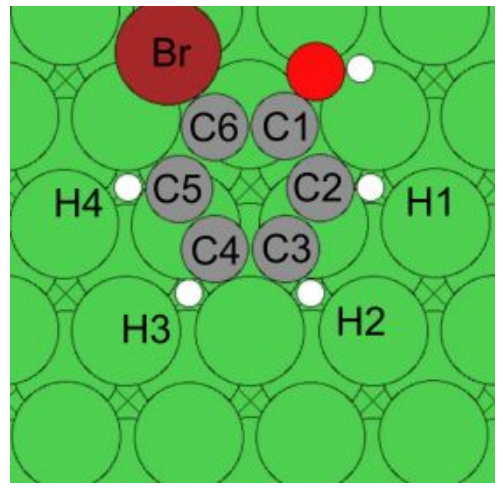
FCC0-2	−0.41	1	H1: 19<br>H2: 16<br>H3: 17<br>H4: 18<br>Br: 23<br>OH: 21	C1−C2: 1.424<br>C2−C3: 1.449<br>C3−C4: 1.419<br>C4−C5: 1.445<br>C5−C6: 1.429<br>C6−C1: 1.457	1.956	2.203	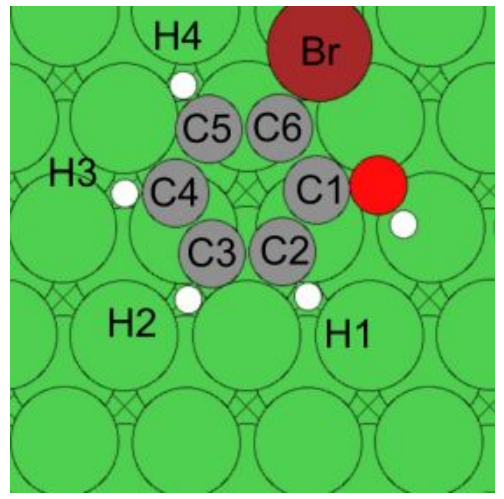

FCC0-3	−0.44	0	H1: 18<br>H2: 17<br>H3: 17<br>H4: 18<br>Br: 24<br>OH: 21	C1−C2: 1.452<br>C2−C3: 1.427<br>C3−C4: 1.443<br>C4−C5: 1.421<br>C5−C6: 1.456<br>C6−C1: 1.425	1.952	2.201	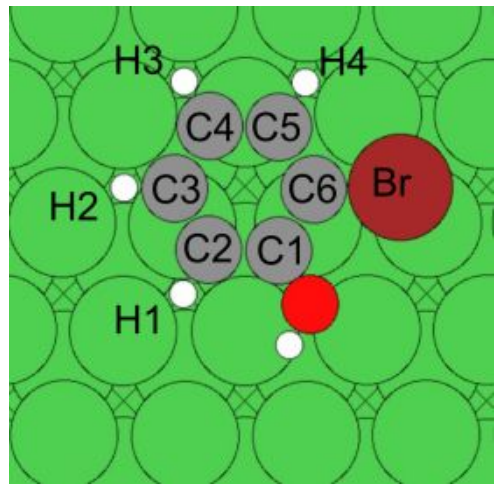
FCC0-4	−0.40	2	H1: 19<br>H2: 16<br>H3: 17<br>H4: 18<br>Br: 23<br>OH: 21	C1−C2: 1.426<br>C2−C3: 1.449<br>C3−C4: 1.419<br>C4−C5: 1.446<br>C5−C6: 1.430<br>C6−C1: 1.454	1.955	2.195	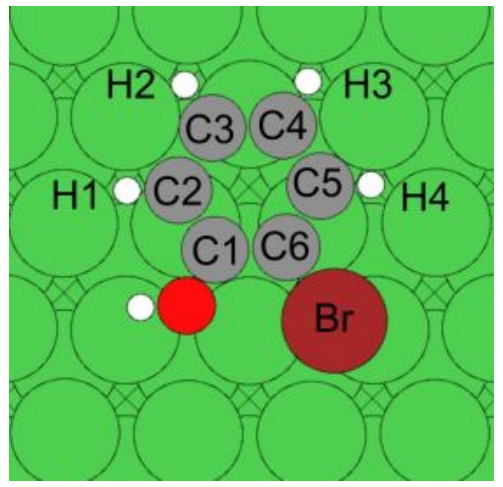
FCC0-5	−0.45	1	H1: 18<br>H2: 17<br>H3: 17<br>H4: 17<br>Br: 23<br>OH: 22	C1−C2: 1.451<br>C2−C3: 1.426<br>C3−C4: 1.444<br>C4−C5: 1.422<br>C5−C6: 1.454<br>C6−C1: 1.428	1.954	2.195	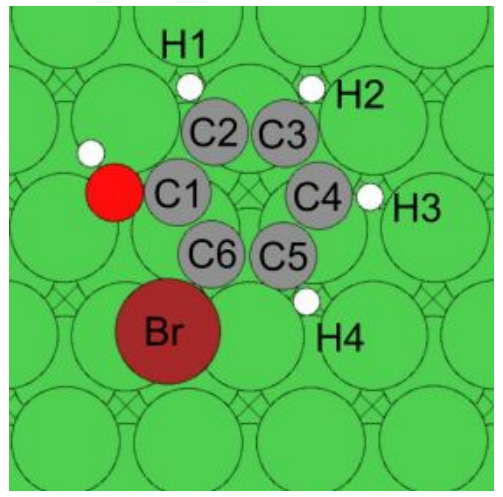
FCC0-6	−0.40	0	H1: 19<br>H2: 17<br>H3: 17<br>H4: 18<br>Br: 23<br>OH: 20	C1−C2: 1.425<br>C2−C3: 1.449<br>C3−C4: 1.418<br>C4−C5: 1.444<br>C5−C6: 1.431<br>C6−C1: 1.454	1.956	2.191	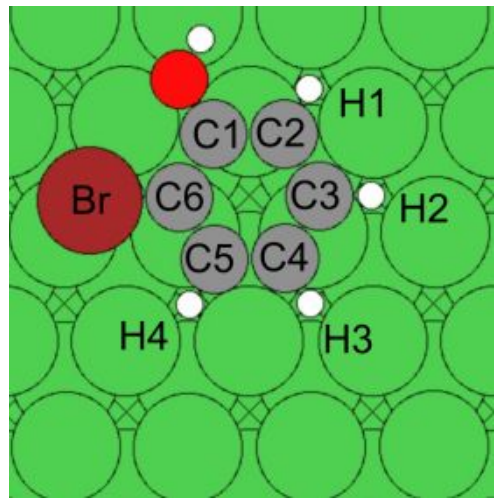

Table S6 Studied close-coordination geometries for 2-bromophenol on HCP hollow site calculated with BEEF-vdW.

Name	E_{ads} (eV)	C-C Ni-Ni angle (°)	Angles (°)	C-C bonds (Å)	C-Br bonds (Å)	Avg Distance from slab (Å)	Optimized geometry
HCP30-1	−0.42	28	H1: 18<br>H2: 12<br>H3: 16<br>H4: 14<br>Br: 24<br>OH: 16	C1−C2: 1.428<br>C2−C3: 1.436<br>C3−C4: 1.425<br>C4−C5: 1.435<br>C5−C6: 1.442<br>C6−C1: 1.431	1.951	2.236	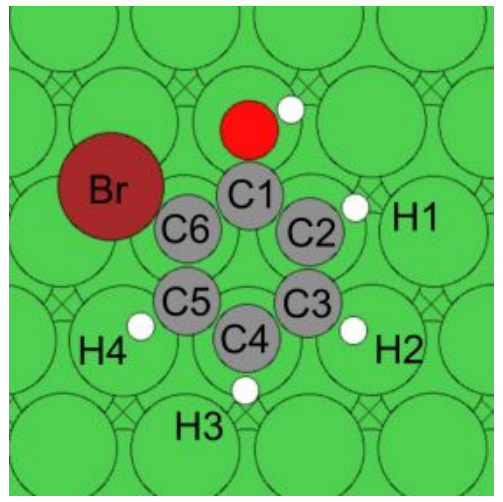
HCP30-2	Saddle point<br>Migrates	-	-	-	-	-	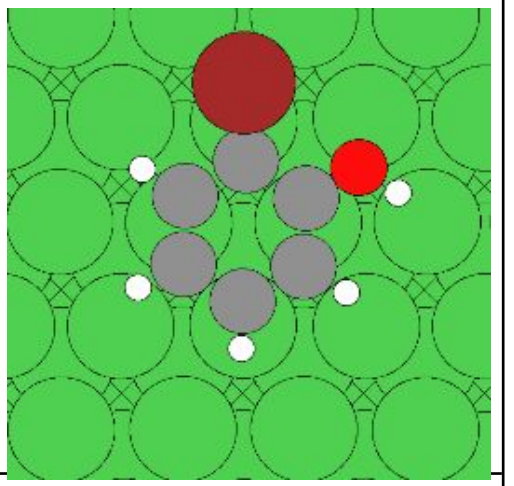
HCP30-3	−0.43	29	H1: 18<br>H2: 12<br>H3: 17<br>H4: 14<br>Br: 24<br>OH: 16	C1−C2: 1.425<br>C2−C3: 1.436<br>C3−C4: 1.427<br>C4−C5: 1.431<br>C5−C6: 1.444<br>C6−C1: 1.435	1.956	2.233	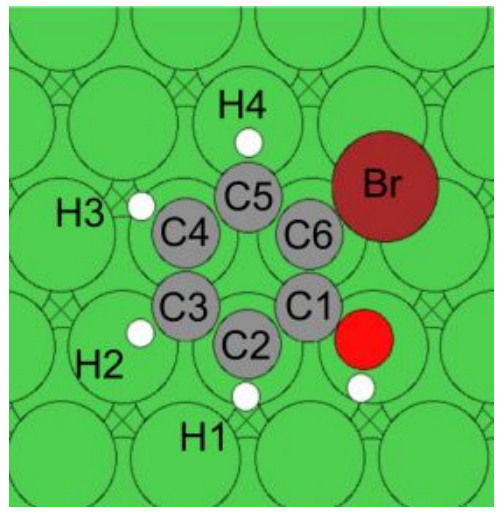
HCP30-4	−0.29	29	H1: 16<br>H2: 18<br>H3: 13<br>H4: 18<br>Br: 23<br>OH: 21	C1−C2: 1.448<br>C2−C3: 1.439<br>C3−C4: 1.430<br>C4−C5: 1.434<br>C5−C6: 1.430<br>C6−C1: 1.425	1.924	2.203	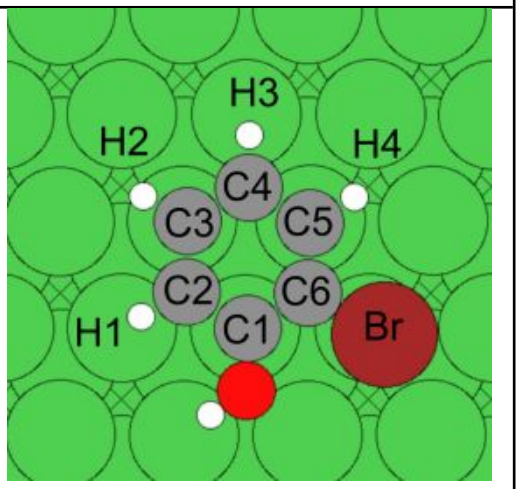

HCP30-5	−0.41	30	H1: 17<br>H2: 13<br>H3: 17<br>H4: 14<br>Br: 24<br>OH: 16	C1−C2: 1.425<br>C2−C3: 1.434<br>C3−C4: 1.428<br>C4−C5: 1.432<br>C5−C6: 1.443<br>C6−C1: 1.435	1.955	2.234	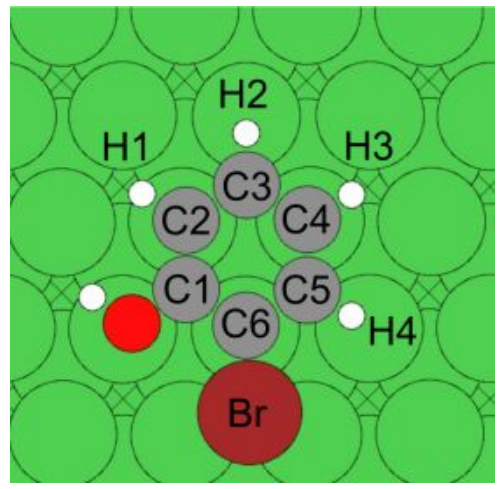
HCP30-6	−0.29	31	H1: 16<br>H2: 18<br>H3: 13<br>H4: 18<br>Br: 23<br>OH: 21	C1−C2: 1.444<br>C2−C3: 1.441<br>C3−C4: 1.431<br>C4−C5: 1.431<br>C5−C6: 1.434<br>C6−C1: 1.428	1.931	2.192	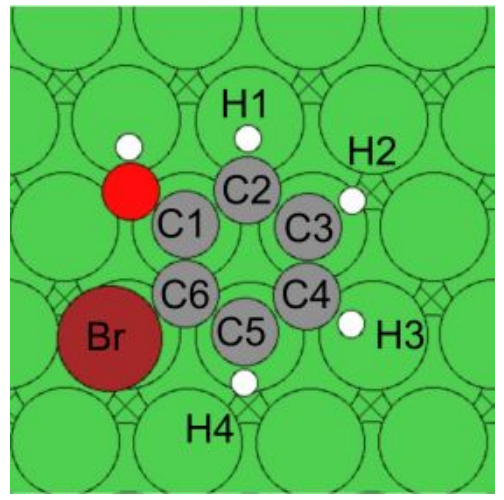
HCP0-1	−0.42	1	H1: 19<br>H2: 16<br>H3: 17<br>H4: 18<br>Br: 23<br>OH: 20	C1−C2: 1.425<br>C2−C3: 1.450<br>C3−C4: 1.419<br>C4−C5: 1.445<br>C5−C6: 1.431<br>C6−C1: 1.455	1.957	2.210	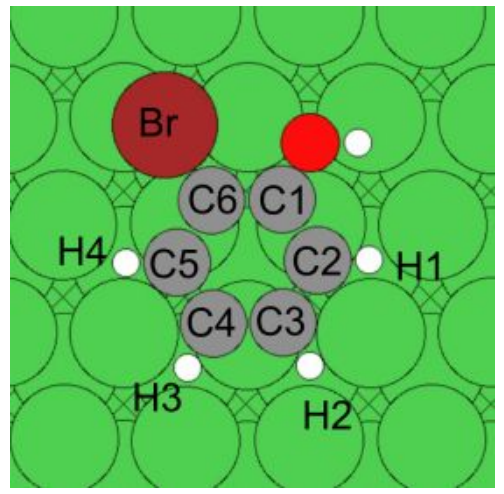
HCP0-2	−0.47	0	H1: 18<br>H2: 17<br>H3: 17<br>H4: 17<br>Br: 24<br>OH: 22	C1−C2: 1.452<br>C2−C3: 1.426<br>C3−C4: 1.443<br>C4−C5: 1.423<br>C5−C6: 1.456<br>C6−C1:1.428	1.955	2.195	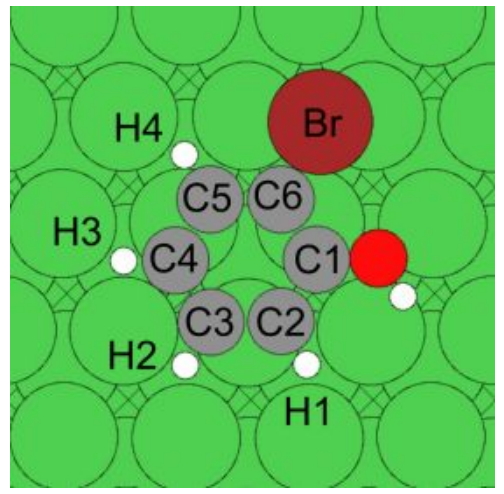

HCP0-3	−0.43	1	H1: 19<br>H2: 17<br>H3: 16<br>H4: 18<br>Br: 24<br>OH: 21	C1−C2: 1.426<br>C2−C3: 1.449<br>C3−C4: 1.419<br>C4−C5: 1.445<br>C5−C6: 1.432<br>C6−C1: 1.455	1.955	2.201	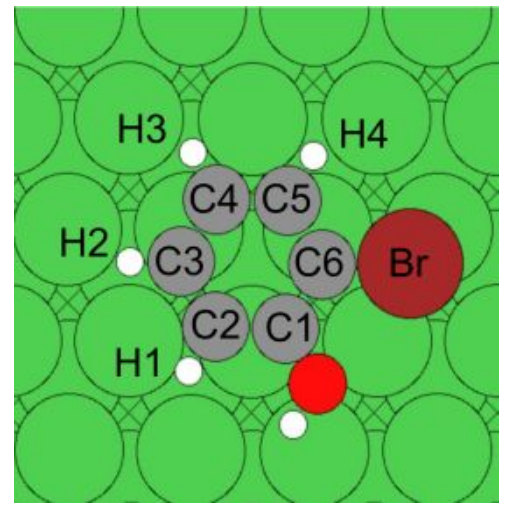
HCP0-4	−0.47	1	H1: 18<br>H2: 17<br>H3: 17<br>H4: 17<br>Br: 24<br>OH: 21	C1−C2: 1.452<br>C2−C3: 1.427<br>C3−C4: 1.444<br>C4−C5: 1.422<br>C5−C6: 1.457<br>C6−C1: 1.426	1.954	2.202	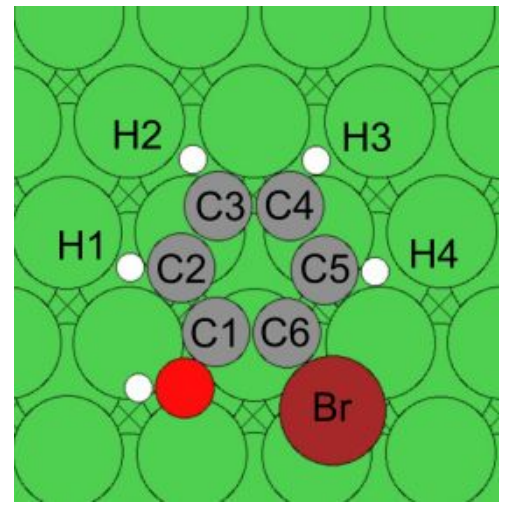
HCP0-5	−0.44	0	H1: 19<br>H2: 17<br>H3: 17<br>H4: 18<br>Br: 23<br>OH: 21	C1−C2: 1.425<br>C2−C3: 1.450<br>C3−C4: 1.419<br>C4−C5: 1.445<br>C5−C6: 1.432<br>C6−C1: 1.456	1.958	2.198	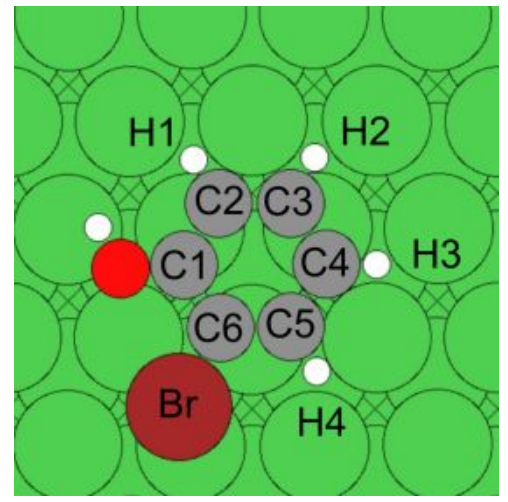
HCP0-6	−0.47	2	H1: 18<br>H2: 17<br>H3: 17<br>H4: 17<br>Br: 23<br>OH: 21	C1−C2: 1.451<br>C2−C3: 1.427<br>C3−C4: 1.444<br>C4−C5: 1.422<br>C5−C6: 1.456<br>C6−C1: 1.424	1.953	2.203	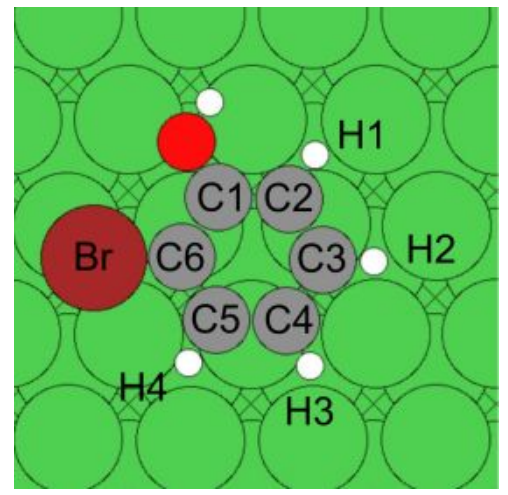

Table S7 Studied close-coordination geometries for phenol on bridge site calculated with BEEF-vdW.

Name	E_{ads} (eV)	C-C Ni-Ni angle (°)	Angles (°)	C-C bonds (Å)	Avg Distance from slab (Å)	Optimized geometry
Bri30-1	−0.57	30	H1: 26<br>H2: 15<br>H3: 14<br>H4: 22<br>H5: 17<br>OH: 20	C1−C2: 1.443<br>C2−C3: 1.452<br>C3−C4: 1.426<br>C4−C5: 1.442<br>C5−C6: 1.448<br>C6−C1: 1.421	2.187	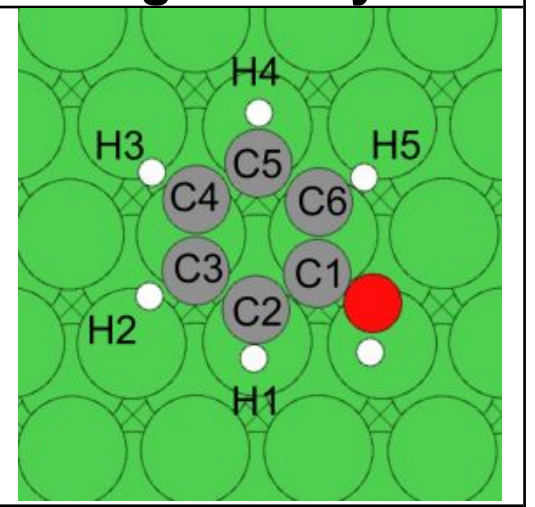
Bri30-2	−0.50	30	H1: 18<br>H2: 15<br>H3: 22<br>H4: 15<br>H5: 18<br>OH: 31	C1−C2: 1.455<br>C2−C3: 1.432<br>C3−C4: 1.441<br>C4−C5: 1.442<br>C5−C6: 1.430<br>C6−C1: 1.446	2.159	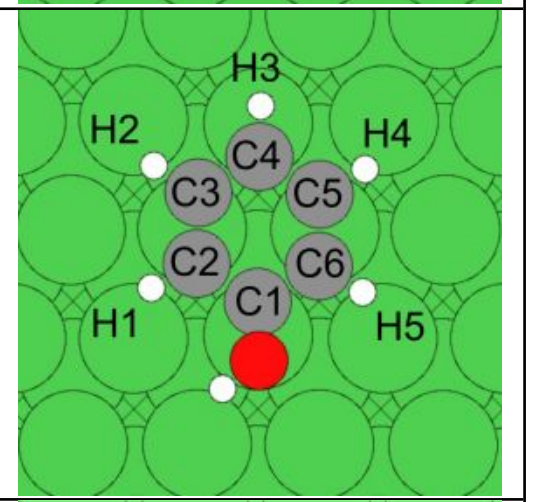
Bri0-1	−0.33	0	H1: 20<br>H2: 18<br>H3: 17<br>H4: 18<br>H5: 20<br>OH: 23	C1−C2: 1.446<br>C2−C3: 1.432<br>C3−C4: 1.438<br>C4−C5: 1.439<br>C5−C6: 1.428<br>C6−C1: 1.442	2.165	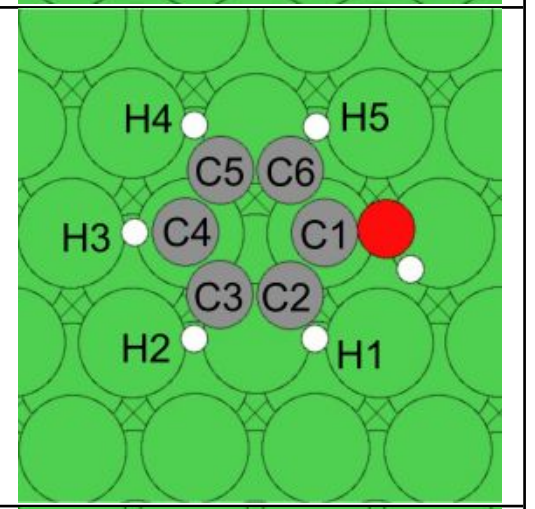
Bri0-2	−0.32	0	H1: 19<br>H2: 17<br>H3: 17<br>H4: 17<br>H5: 18<br>OH: 20	C1−C2: 1.424<br>C2−C3: 1.448<br>C3−C4: 1.440<br>C4−C5: 1.423<br>C5−C6: 1.444<br>C6−C1: 1.430	2.196	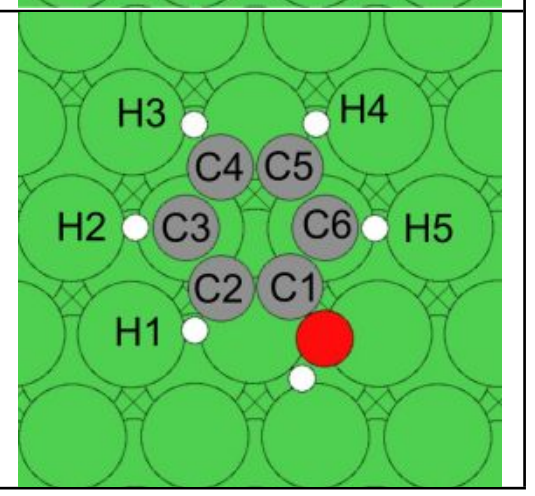

Table S8 Studied close-coordination geometries for phenol on FCC hollow site calculated with BEEF-vdW.

Name	E_{ads} (eV)	C-C Ni-Ni angle (°)	Angles (°)	C-C bonds (Å)	Avg Distance from slab (Å)	Optimized geometry
FCC30-1	−0.37	29	H1: 15<br>H2: 17<br>H3: 14<br>H4: 16<br>H5: 15<br>OH: 23	C1−C2: 1.444<br>C2−C3: 1.434<br>C3−C4: 1.431<br>C4−C5: 1.430<br>C5−C6: 1.432<br>C6−C1: 1.431	2.204	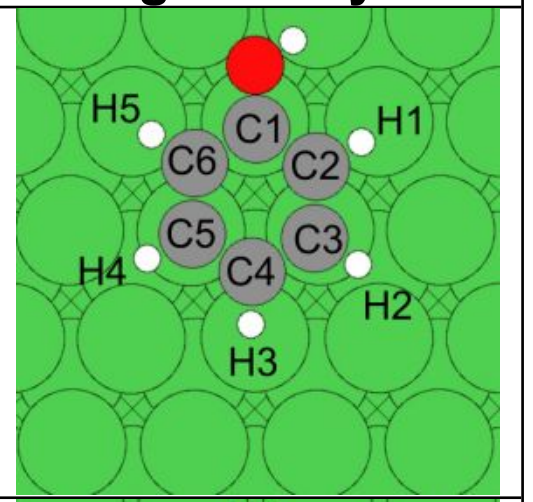
FCC30-2	−0.38	30	H1: 15<br>H2: 17<br>H3: 13<br>H4: 17<br>H5:15<br>OH: 21	C1−C2: 1.436<br>C2−C3: 1.437<br>C3−C4: 1.430<br>C4−C5: 1.427<br>C5−C6: 1.433<br>C6−C1: 1.431	2.214	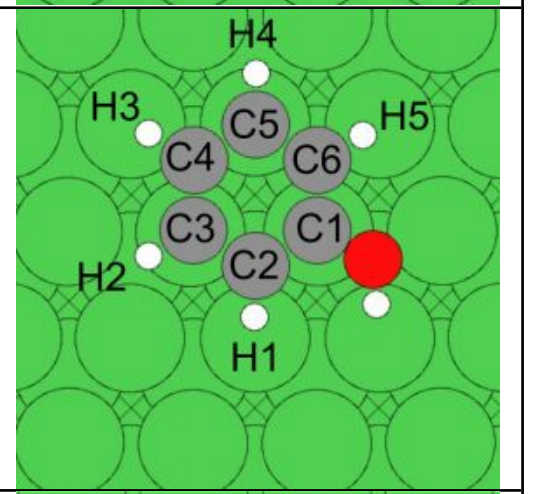
FCC30-3	−0.44	31	H1: 17<br>H2: 11<br>H3: 15<br>H4: 11<br>H5:16<br>OH: 15	C1−C2: 1.426<br>C2−C3: 1.433<br>C3−C4: 1.429<br>C4−C5: 1.428<br>C5−C6: 1.430<br>C6−C1: 1.422	2.258	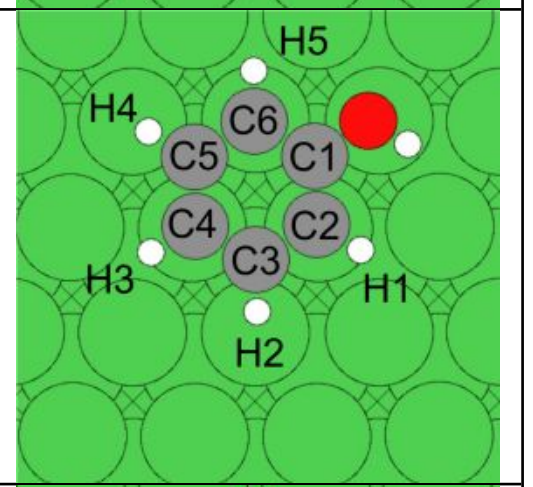
FCC30-4	−0.45	29	H1: 17<br>H2: 11<br>H3: 15<br>H4: 11<br>H5:16<br>OH: 15	C1−C2: 1.426<br>C2−C3: 1.435<br>C3−C4: 1.427<br>C4−C5: 1.427<br>C5−C6: 1.431<br>C6−C1: 1.421	2.256	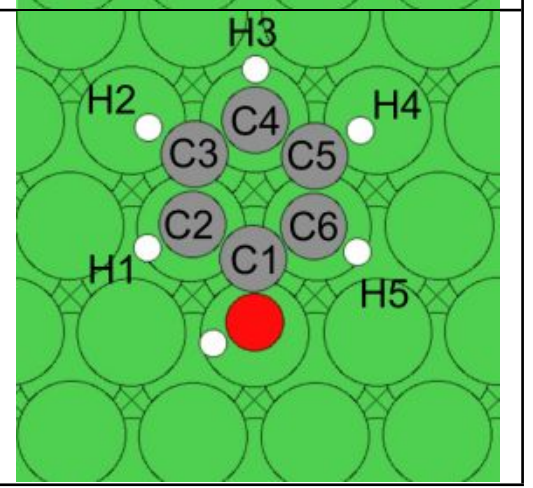

FCC0-1	−0.51	0	H1: 18<br>H2: 16<br>H3: 16<br>H4: 16<br>H5:18<br>OH: 21	C1−C2: 1.450<br>C2−C3: 1.428<br>C3−C4: 1.444<br>C4−C5: 1.421<br>C5−C6: 1.449<br>C6−C1: 1.417	2.213	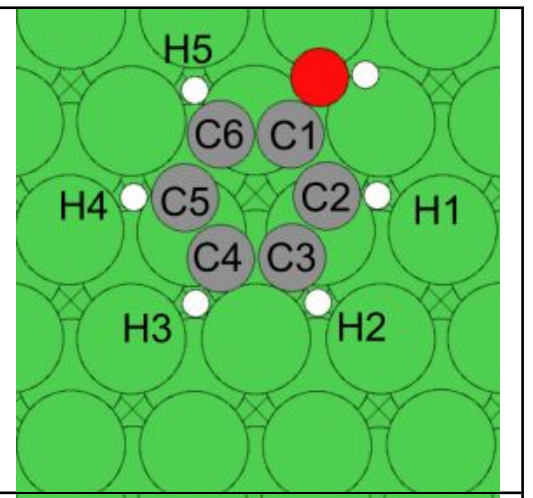
FCC0-2	−0.49	0	H1: 18<br>H2: 16<br>H3: 16<br>H4: 16<br>H5:17<br>OH: 20	C1−C2: 1.420<br>C2−C3: 1.450<br>C3−C4: 1.422<br>C4−C5: 1.444<br>C5−C6: 1.423<br>C6−C1: 1.444	2.211	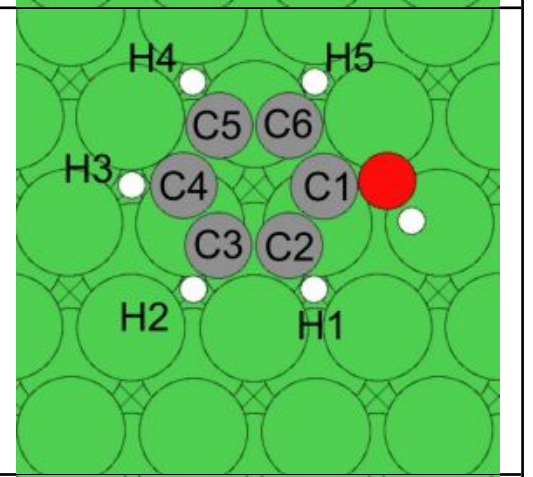
FCC0-3	−0.51	0	H1: 18<br>H2: 16<br>H3: 16<br>H4: 16<br>H5:18<br>OH: 21	C1−C2: 1.448<br>C2−C3: 1.429<br>C3−C4: 1.445<br>C4−C5: 1.419<br>C5−C6: 1.450<br>C6−C1: 1.417	2.211	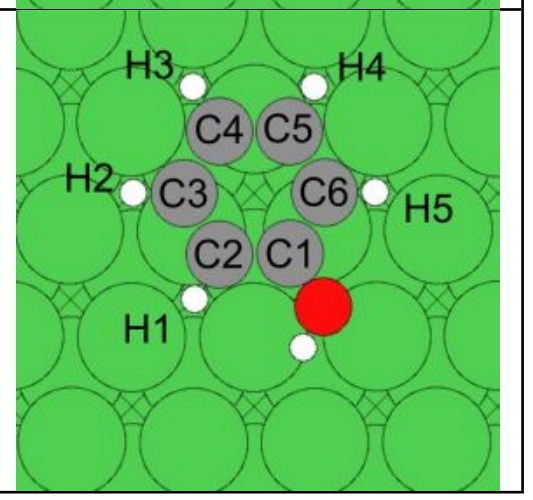

Table S9 Studied close-coordination geometries for phenol on HCP hollow site calculated with BEEF-vdW.

Name	E_{ads} (eV)	C-C Ni-Ni angle (°)	Angles (°)	C-C bonds (Å)	Avg Distance from slab (Å)	Optimized geometry
HCP30-1	−0.43	31	H1: 16<br>H2: 17<br>H3: 14<br>H4: 16<br>H5:16<br>OH: 22	C1−C2: 1.437<br>C2−C3: 1.439<br>C3−C4: 1.431<br>C4−C5: 1.430<br>C5−C6: 1.434<br>C6−C1: 1.433	2.179	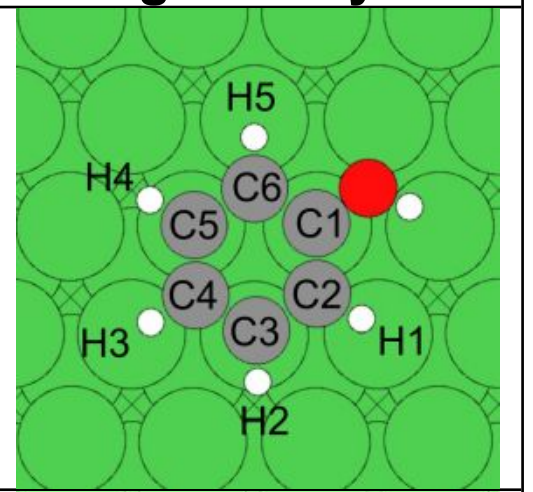
HCP30-2	−0.43	30	H1: 16<br>H2: 17<br>H3: 13<br>H4: 17<br>H5:16<br>OH: 22	C1−C2: 1.440<br>C2−C3: 1.438<br>C3−C4: 1.429<br>C4−C5: 1.431<br>C5−C6: 1.435<br>C6−C1: 1.430	2.185	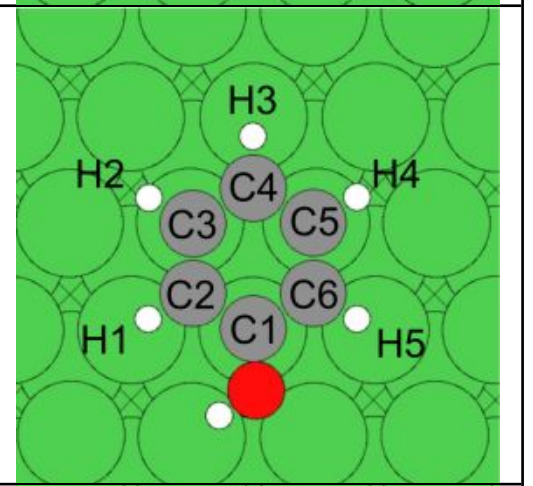
HCP30-3	−0.49	27	H1: 18<br>H2: 11<br>H3: 16<br>H4: 11<br>H5: 16<br>OH: 15	C1−C2: 1.425<br>C2−C3: 1.438<br>C3−C4: 1.426<br>C4−C5: 1.430<br>C5−C6: 1.431<br>C6−C1: 1.422	2.258	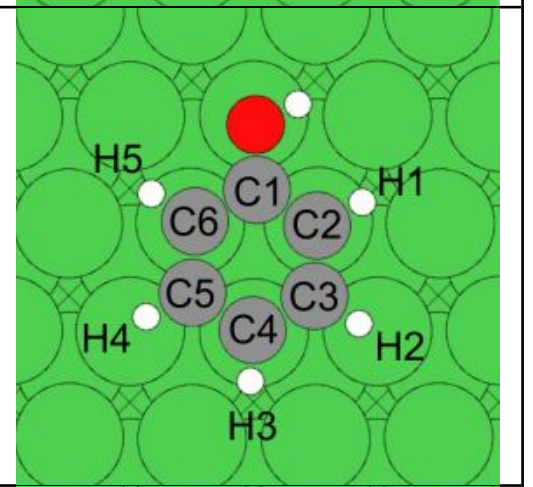
HCP30-4	−0.49	30	H1: 17<br>H2: 11<br>H3: 15<br>H4: 12<br>H5: 17<br>OH: 16	C1−C2: 1.424<br>C2−C3: 1.437<br>C3−C4: 1.429<br>C4−C5: 1.426<br>C5−C6: 1.434<br>C6−C1: 1.424	2.254	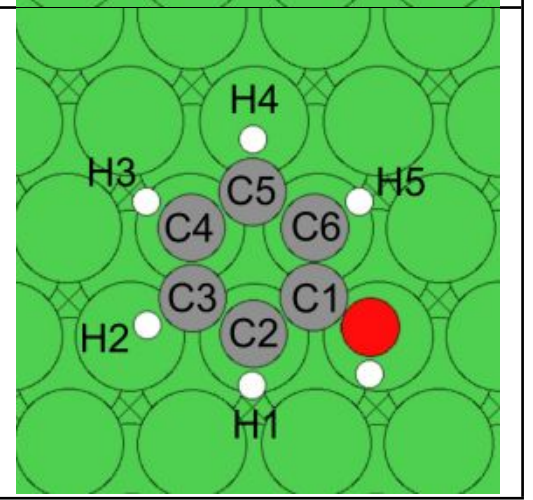

HCP0-1	−0.52	1	H1: 19<br>H2: 16<br>H3: 16<br>H4: 16<br>H5: 17<br>OH: 20	C1−C2: 1.421<br>C2−C3: 1.452<br>C3−C4: 1.420<br>C4−C5: 1.444<br>C5−C6: 1.424<br>C6−C1: 1.443	2.219	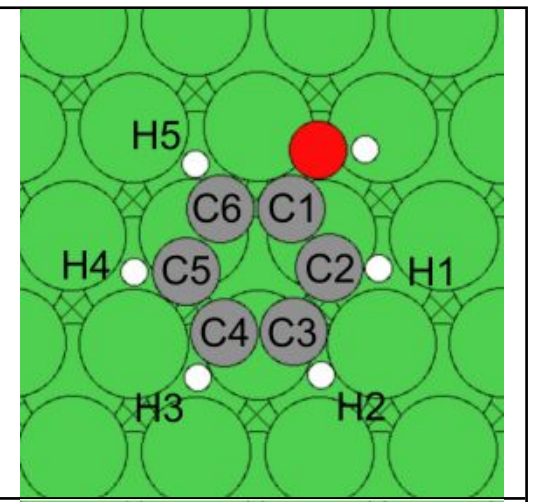
HCP0-2	−0.55	1	H1: 18<br>H2: 16<br>H3: 16<br>H4: 16<br>H5:18<br>OH: 21	C1−C2: 1.449<br>C2−C3: 1.428<br>C3−C4: 1.445<br>C4−C5: 1.421<br>C5−C6: 1.449<br>C6−C1: 1.419	2.206	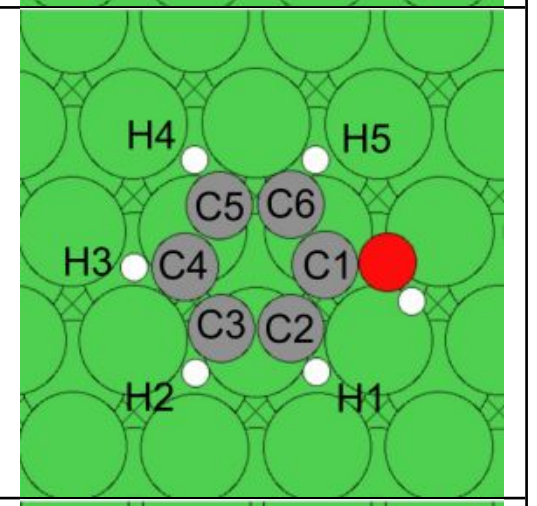
HCP0-3	−0.52	0	H1: 18<br>H2: 16<br>H3: 15<br>H4: 16<br>H5: 18<br>OH: 20	C1−C2: 1.420<br>C2−C3: 1.452<br>C3−C4: 1.420<br>C4−C5: 1.445<br>C5−C6: 1.425<br>C6−C1: 1.444	2.216	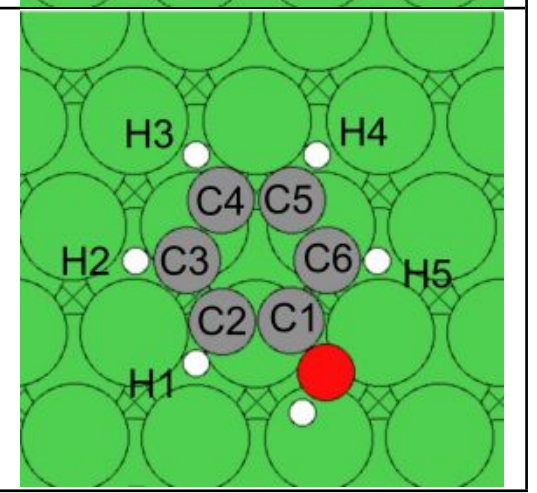

Table S10 Bader charge values (|e|) for high coordination (HC) and close coordination (CC) 2,6-dibromophenol on Ni(111) surface. Negative charge corresponds to deficiency, and positive excess of electrons relative to isolated atoms or optimized ensemble. In the latter situation nickel atoms are compared to bader chargers of isolated Ni(111), while other atoms are compared to isolated gas-phase 2,6-dibromophenol. Difference is calculated by subtracting CC values from HC values.

	Relative to isolated atoms		Relative to optimized structures		Difference
	DBP HC	DBP CC	DBP HC	DBP CC	
Ni	0.27	-0.27	-0.04	-0.57	0.53
C	-0.47	0.03	0	0.52	-0.52
H	-0.14	-0.22	-0.01	-0.10	0.09
Br	0.13	0.21	-0.01	0.07	-0.08
OH	0.52	0.48	0.02	-0.02	0.04

Table S11 Bader charge values (|e|) for first re-coordination of 2,6-dibromophenol from high coordination to close coordination geometry (**1HC → 1CC**). Negative charge corresponds to deficiency, and positive excess of electrons relative to isolated atoms.

	**Initial 1**	**2**	**3**	**4**	**TS 5**	**6**	**Final 7**
**Nickel (top layer)**	0.248	0.250	0.246	0.248	0.092	-0.148	-0.263
**Carbon ring**	-0.467	-0.448	-0.425	-0.398	-0.239	-0.071	0.001
**Hydrogens (ring)**	-0.143	-0.166	-0.157	-0.173	-0.229	-0.246	-0.206
**Hydrogen (hydroxyl)**	-0.574	-0.581	-0.580	-0.578	-0.588	-0.580	-0.574
**Bromine**	0.123	0.134	0.117	0.108	0.114	0.170	0.210
**Oxygen**	1.087	1.091	1.071	1.068	1.082	1.073	1.050

Table S12 Bader charge values (|e|) for first debromination step of 2,6-dibromophenol. Negative charge corresponds to deficiency, and positive excess of electrons relative to isolated atoms.

**NEB image**	**Initial 1**	**2**	**3**	**TS 4**	**5**	**6**	**7**	**8**	**Final 9**
**Nickel (top layer)**	-0.266	-0.309	-0.367	-0.455	-0.556	-0.614	-0.661	-0.694	-0.673
**Carbon ring**	0.002	0.055	0.027	0.026	0.113	0.146	0.171	0.178	0.208
**Hydrogens (ring)**	-0.206	-0.240	-0.206	-0.199	-0.253	-0.237	-0.206	-0.179	-0.233
**Hydrogen (hydroxyl)**	-0.567	-0.580	-0.586	-0.585	-0.593	-0.584	-0.589	-0.590	-0.571
**Bromine**	0.209	0.243	0.306	0.396	0.471	0.496	0.494	0.470	0.459
**Bromine (detaching)**	0.149	0.185	0.259	0.362	0.447	0.478	0.471	0.438	0.436
**Oxygen**	1.050	1.056	1.060	1.062	1.068	1.054	1.055	1.061	1.047

Table S13 Bader charge values (|e|) for first hydrogenation step after first debromination. Negative charge corresponds to deficiency, and positive excess of electrons relative to isolated atoms.

NEB image	Initial 1	2	3	4	TS 5	6	7	8	Final 9
Nickel (top layer)	-0.406	-0.326	-0.265	-0.177	-0.076	0.134	0.243	0.252	0.253
Carbon ring	-0.145	-0.158	-0.182	-0.196	-0.240	-0.301	-0.388	-0.413	-0.445
Hydrogens (ring)	-0.085	-0.106	-0.116	-0.133	-0.122	-0.160	-0.132	-0.114	-0.108
Hydrogen (hydroxyl)	-0.574	-0.571	-0.568	-0.570	-0.565	-0.566	-0.571	-0.563	-0.566
Hydrogen (attatching)	0.257	0.222	0.169	0.115	0.056	-0.034	-0.036	-0.045	-0.036
Oxygen	1.087	1.086	1.079	1.083	1.077	1.081	1.076	1.082	1.082
Bromine	0.094	0.095	0.094	0.094	0.092	0.082	0.086	0.091	0.099

Table S14 Bader charge values (|e|) for direct hydrogenolysis of 2,6-dibromophenol. Negative charge corresponds to excess, and positive charge to deficiency of electrons relative to isolated atoms.

NEB image	Initial 1	2	3	4	TS 5	6	7	8	Final 9
Nickel (top layer)	-0.519	-0.490	-0.447	-0.396	-0.347	-0.259	-0.296	-0.423	-0.544
Carbon ring	0.043	0.023	0.004	0.019	0.021	-0.026	-0.059	0.067	0.045
Hydrogens (ring)	-0.237	-0.222	-0.194	-0.200	-0.207	-0.189	-0.200	-0.239	-0.214
Hydrogen (hydroxyl)	-0.561	-0.551	-0.566	-0.563	-0.566	-0.573	-0.565	-0.566	-0.575
Hydrogen (attatching)	0.251	0.224	0.159	0.101	0.045	-0.041	-0.113	-0.177	-0.090
Bromines	0.185	0.184	0.205	0.219	0.240	0.297	0.494	0.603	0.524
Bromine (detaching)	0.097	0.097	0.112	0.127	0.150	0.217	0.454	0.597	0.443
Oxygen	1.040	1.030	1.046	1.035	1.036	1.048	1.033	1.043	1.067

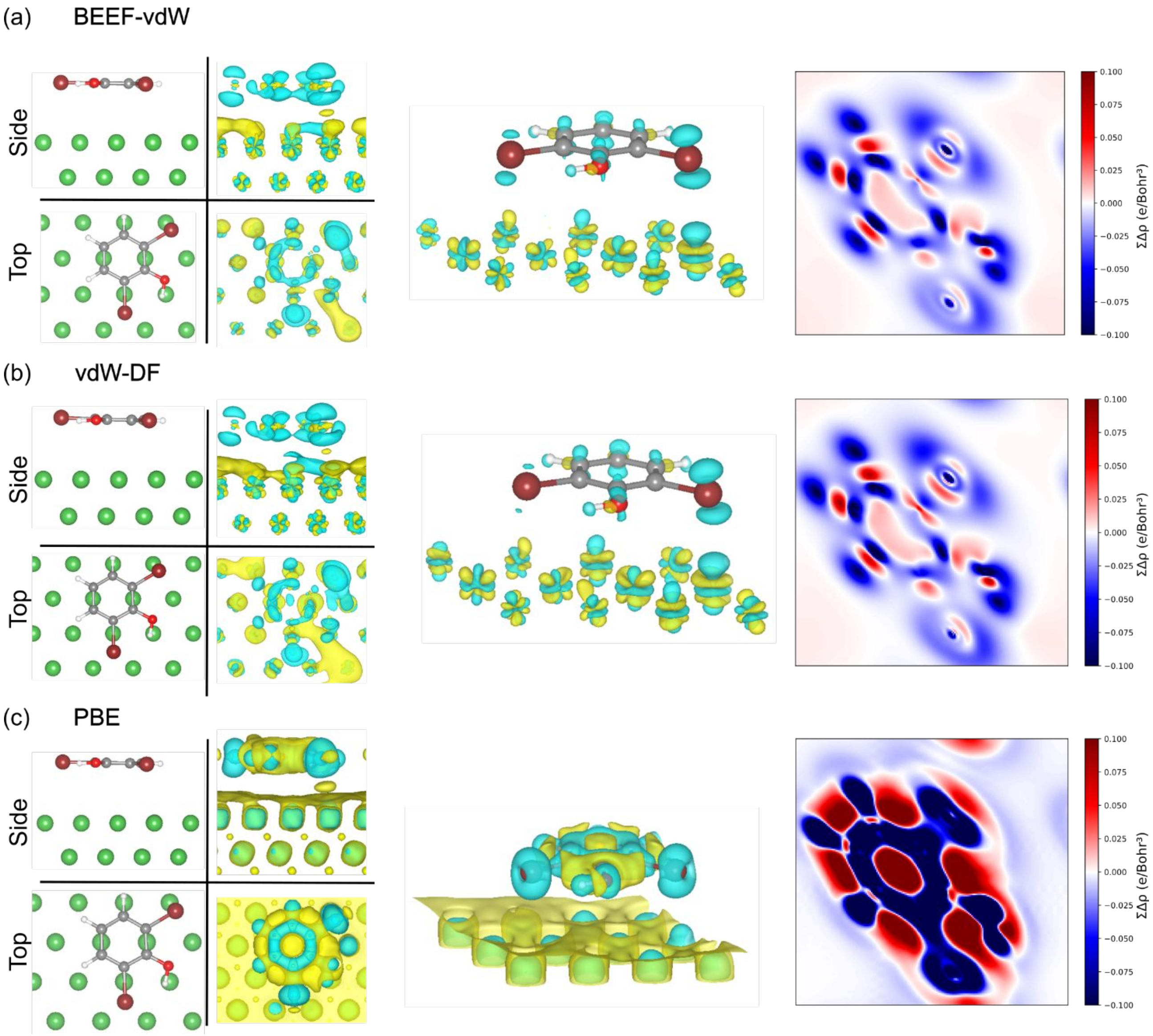

Fig. S1. Charge density difference plots for 2,6-dibromophenol (DBP) over a Ni(111) bridge site produced using (a) BEEF-vdW, (b) vdW-DF, and (c) PBE functionals. Cyan (blue) regions correspond to electron density depletion and yellow (red) regions to electron density accumulation. The left-hand figures present charge density difference isosurfaces at 0.0025 e/Å^3, the middle figures at 0.005 e/Å^3, and the right-hand figures show an xy-slice of the density at the height of the adsorbate.

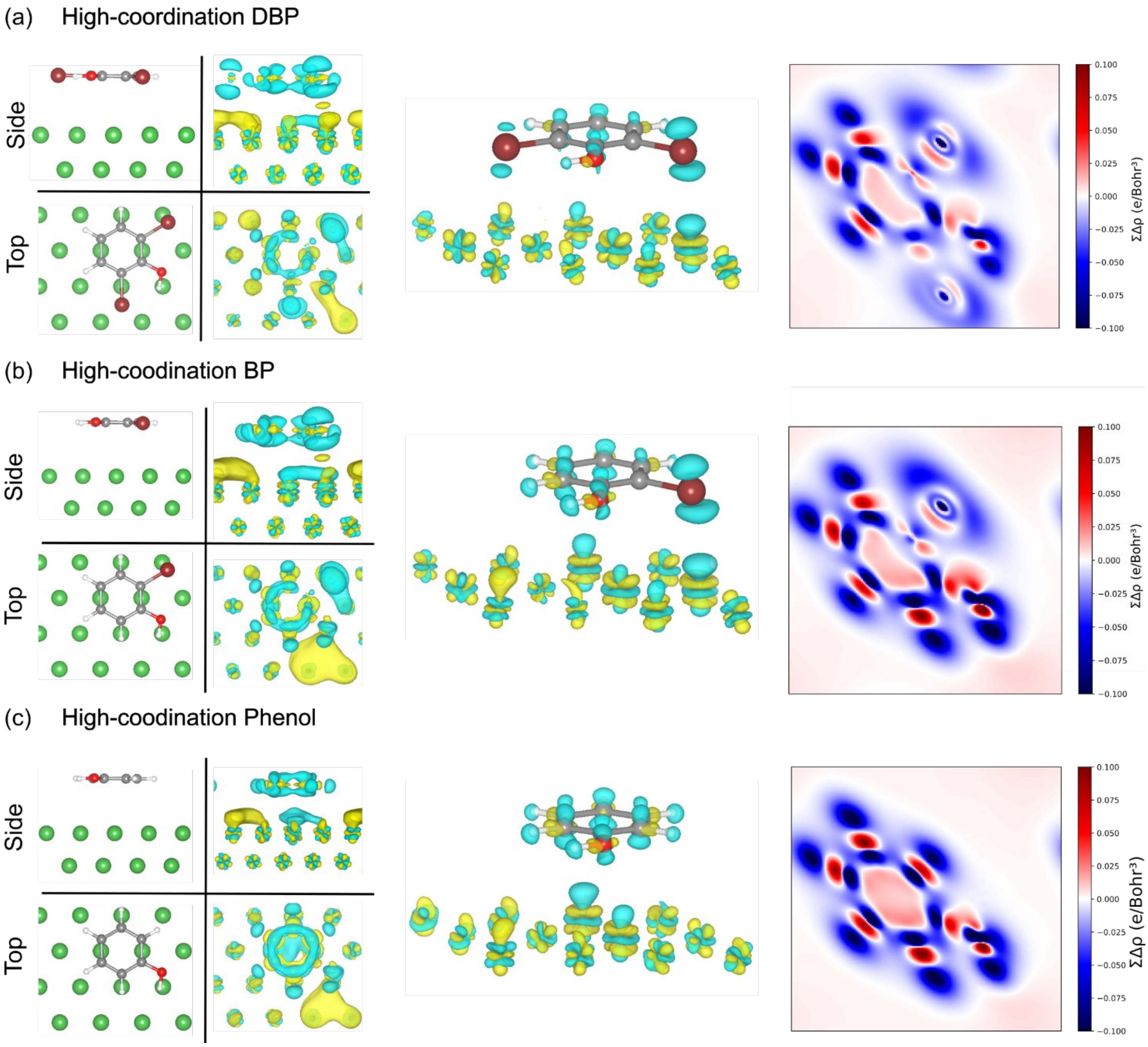

Fig. S2. Charge density difference plots for 2,6-dibromophenol (DBP), 2-bromophenol (BP), and phenol adsorbed in high-coordination geometry over a Ni(111) bridge site. Cyan (blue) regions correspond to electron density depletion and yellow (red) regions to electron density accumulation. The left-hand figures present charge density difference isosurfaces at 0.0025 e/Å^3, the middle figures at 0.005 e/Å^3, and the right-hand figures show an xy-slice of the density at the height of the adsorbate. Bromine substituents in DBP and BP distribute electron density to a larger area, resulting in a more spatially diffuse dipole. In turn, phenol shows the strongest depletion of π-system, indicating that polarization is localized primarily within the ring. In combination with adsorption energy calculations, the results indicate that bromines substitution promotes extensively distributed polarization, which enhances long-range stabilization through dispersion forces.

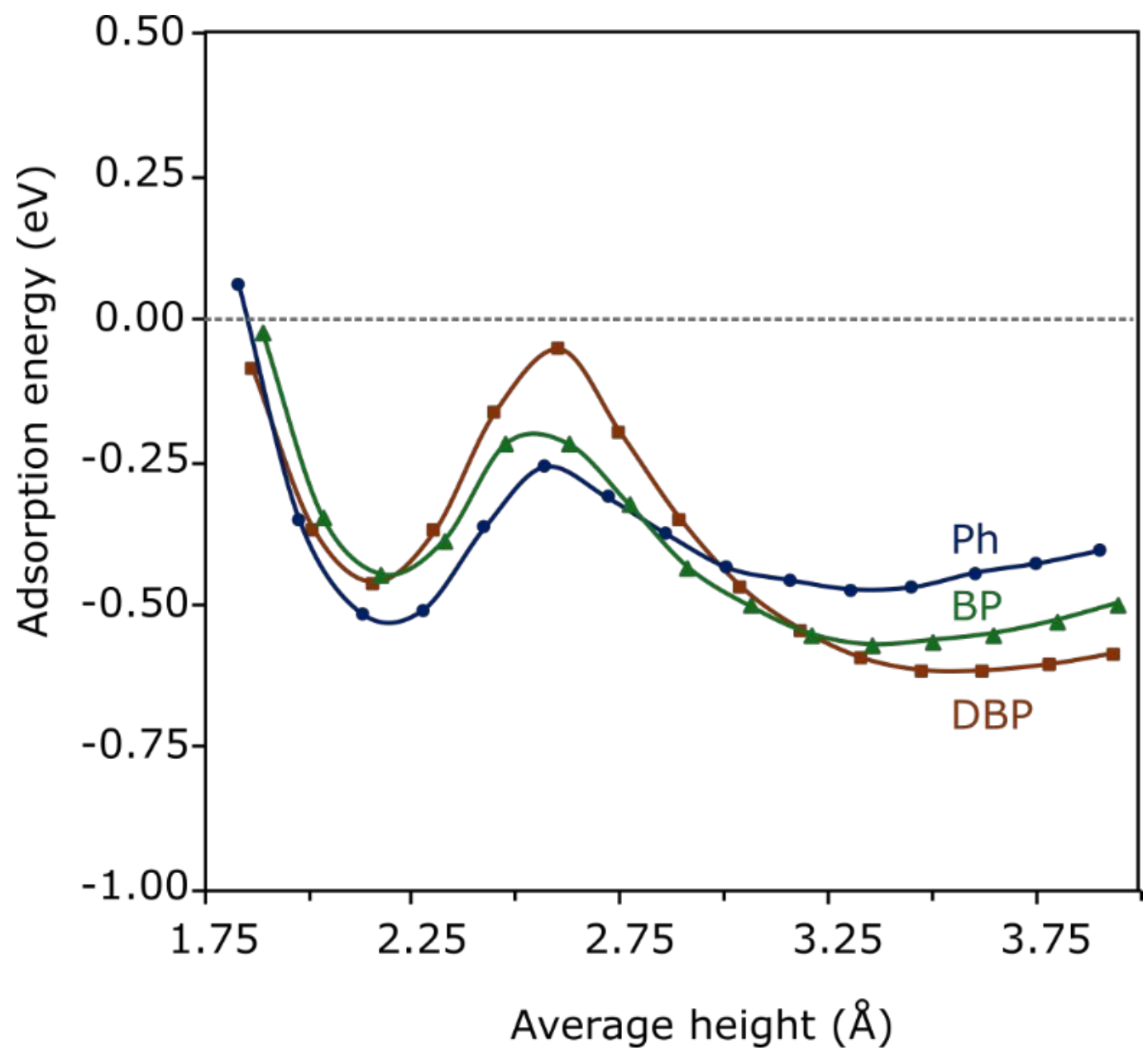

Fig. S3. Height-scan for the 2,6-dibromophenol (DBP), 2-bromophenol (BP) and phenol (Ph) on Ni(111) surface using a BEEF-vdW functional. In scan calculations, positions of every carbon atom were fixed. Hydroxyl group was oriented in the same direction for each adsorbate, and hence the most stable close-coordination geometry was not used for BP.

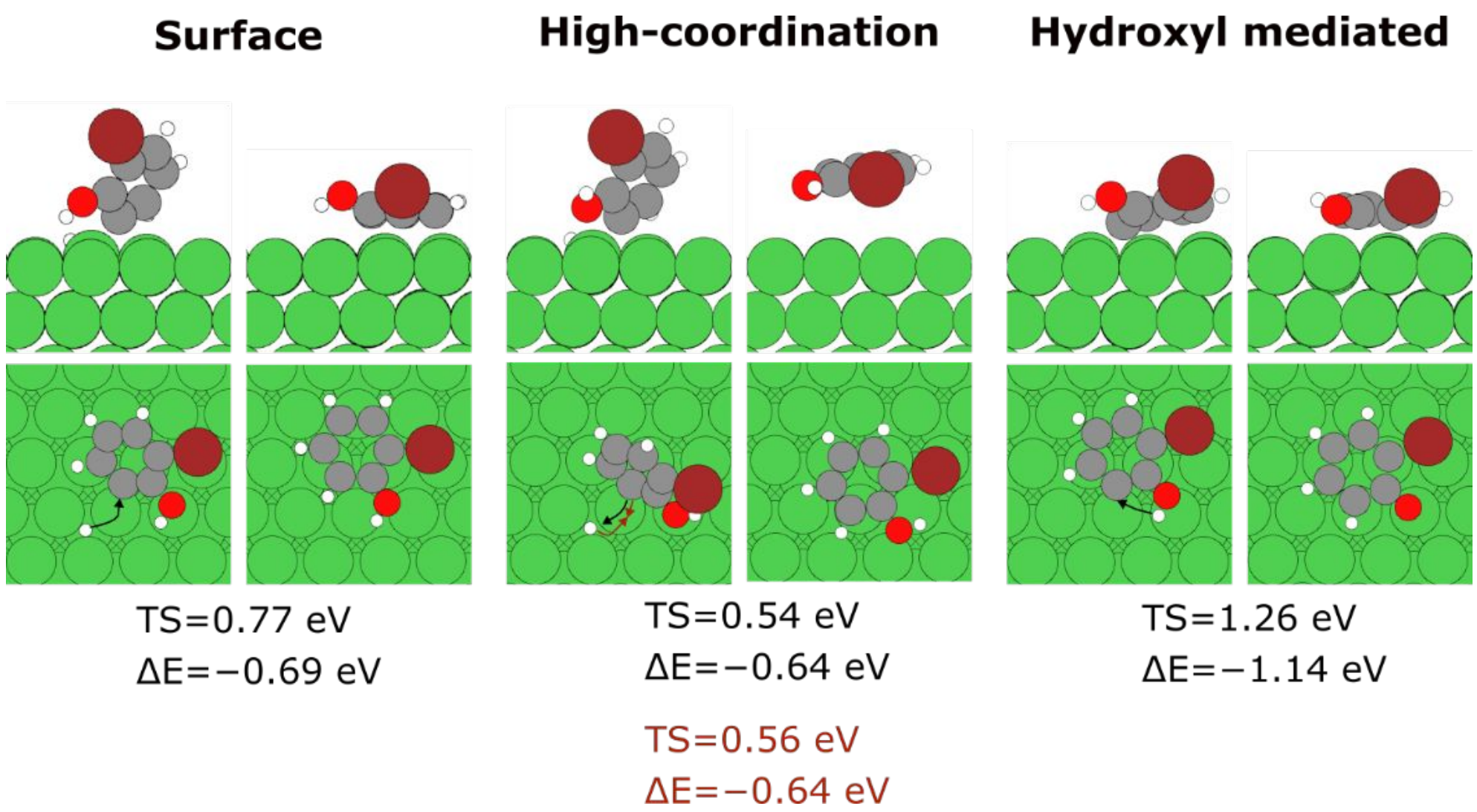

Fig. S4. Studied hydrogenation pathways following the initial debromination step. Energies are presented without zero-point energy-correction. Hydrogenation ending in a close-coordination adsorption geometry involved an activation barrier of 0.77 eV. Two hydrogenation pathways leading to a high-coordination geometry were investigated: one in which aromatic ring moved towards the hydrogen atom and one in which the hydrogen approached the ring. Both paths exhibited practically identical activation barriers of 0.54 eV and 0.56 eV, respectively. The orientation of the hydroxyl group did not affect the barrier heights but resulted in a slight stabilization of the overall energy. In contrast, hydrogen transfer from the hydroxyl group involved a significantly higher activation, but it also led to substantial energetic stabilization due to recovery of aromaticity.

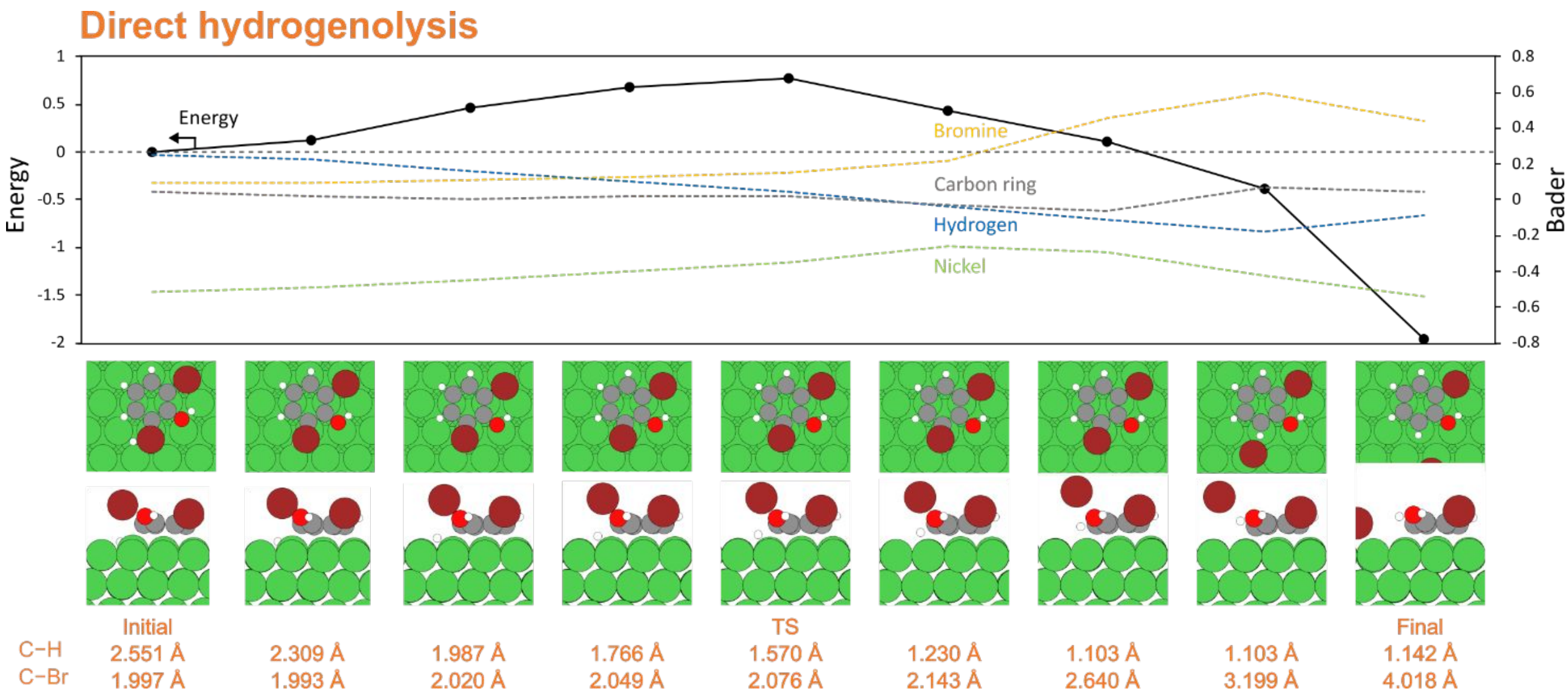

Fig. S5 Minimum energy path (presented without ZPE correction) and associated changes in Bader charges of direct hydrogenolysis of 2,6-dibromophenol.
